# Functions for fission yeast splicing factors SpSlu7 and SpPrp18 in alternative splice-site choice and stress-specific regulated splicing

**DOI:** 10.1371/journal.pone.0188159

**Published:** 2017-12-13

**Authors:** Geetha Melangath, Titash Sen, Rakesh Kumar, Pushpinder Bawa, Subha Srinivasan, Usha Vijayraghavan

**Affiliations:** 1 Department of Microbiology and Cell Biology, Indian Institute of Science, Bangalore, Karnataka, India; 2 Institute of Bioinformatics and Applied Biotechnology, Bangalore, Karnataka, India; Saint George’s University, UNITED KINGDOM

## Abstract

Budding yeast spliceosomal factors ScSlu7 and ScPrp18 interact and mediate intron 3’ss choice during second step pre-mRNA splicing. The fission yeast genome with abundant multi-intronic transcripts, degenerate splice signals and SR proteins is an apt unicellular fungal model to deduce roles for core spliceosomal factors in alternative splice-site choice, intron retention and to study the cellular implications of regulated splicing. From our custom microarray data we deduce a stringent reproducible subset of *S*. *pombe* alternative events. We examined the role of factors SpSlu7 or SpPrp18 for these splice events and investigated the relationship to growth phase and stress. Wild-type log and stationary phase cells showed *ats1*+ exon 3 skipped and intron 3 retained transcripts. Interestingly the non-consensus 5’ss in *ats1+* intron 3 caused SpSlu7 and SpPrp18 dependent intron retention. We validated the use of an alternative 5’ss in *dtd1*+ intron 1 and of an upstream alternative 3’ss in DUF3074 intron 1. The *dtd1*+ intron 1 non-canonical 5’ss yielded an alternative mRNA whose levels increased in stationary phase. Utilization of *dtd1+* intron 1 sub-optimal 5’ ss required functional SpPrp18 and SpSlu7 while compromise in SpSlu7 function alone hampered the selection of the DUF3074 intron 1 non canonical 3’ss. We analysed the relative abundance of these splice isoforms during mild thermal, oxidative and heavy metal stress and found stress-specific splice patterns for *ats1+* and DUF3074 intron 1 some of which were SpSlu7 and SpPrp18 dependent. By studying *ats1+* splice isoforms during compromised transcription elongation rates in wild-type, *spslu7-2* and *spprp18-5* mutant cells we found dynamic and intron context-specific effects in splice-site choice. Our work thus shows the combinatorial effects of splice site strength, core splicing factor functions and transcription elongation kinetics to dictate alternative splice patterns which in turn serve as an additional recourse of gene regulation in fission yeast.

## Introduction

Nuclear pre-mRNA splicing, a key process in eukaryotic gene expression, is carried out by the spliceosome consisting of U1, U2, U4, U5 and U6 snRNAs and ~ 150 non-snRNP proteins. This riboprotein machinery assembles on the pre-mRNA and catalyses the two invariant transesterification reactions by which introns are precisely excised and exons ligated [1]. The flexibility in splice-site choice, reflected as alternative splicing, is crucial for for diversifying the metazoan transcriptome, the proteome and for genome evolution [2, 3]. Extensive tissue- and developmental-stage specific alternative splicing, predominantly exon skipping, is noted in the human transcriptome [4, 5]. In *D*. *melanogaster* and *C*. *elegans*, 25% and 60% respectively of protein coding transcripts undergo alternative splicing [6–8]. Although functions for SR proteins in alternative splice-site choice are well described, roles for core spliceosomal proteins are less understood even in these organisms.

Several features in the fission yeast genome facilitate alternative splicing, these include an abundance of multi-intronic transcripts with degenerate splice-sites, exonic splicing enhancer sequences (ESEs) and the presence of serine/arginine (SR) family of proteins [9–11]. The short introns in fission yeast transcripts resemble introns in other fungal, plant and worm genomes, and such introns are identified and spliced by intron definition [12, 13]. Presence of intron retained forms of fission yeast *zas1*+ and *mes1*+ transcripts in wild-type fission yeast cells together with the suppression of *rem1*+ intron retention during meiosis alone indicate that selective intron retention occurs in fission yeast [14, 15]. Comparative deep sequenced transcriptomes of several fission yeast species confirm that intron retention is the most common alternative transcript form, while transcript isoforms generated by the use of alternative splice-donor or splice-acceptor sites in a given intron are comparatively less frequent [16]. A refined approach of deep sequencing of cellular RNA species enriched as intron lariats in the *S*. *pombe spdbr1Δ* genetic knockout of lariat debranchase enzyme [17, 18] identified new splicing events including several exon skipping events. Furthermore, extensive analyses of the transcriptome data in mutant strains for various RNA processing mutants and of wild-type cells subjected to various stresses [19, 20] indicate that the use of altered splice sites is more rampant than previously reported for fission yeast. These events are thought to yield aberrant transcripts that are likely substrates for RNA surveillance machineries [19, 21]. Interestingly, genome-wide comparison of alternate splicing events across 23 fungal species showed higher propensity of alternative splicing in genes governing virulence, stress response and dimorphic switching [22].

While alternative splicing is rare in budding yeast, several splicing factors influence splice-site selection in the few instances of alternative splicing that are known. The use of a non-canonical 5’ss in *SRC1* single intron requires non-covalent interaction of budding yeast ScHub1, an ubiquitin like protein, with Snu66, a tri-snRNP member [23]. Similarly, use of this sub-optimal donor splice-site does not occur in cells lacking ScBud31, a nineteen complex (NTC) member [24]. In vitro splicing with model intron containing transcripts and other transcriptome sequencing studies show that budding yeast second-step splicing factors ScSlu7, ScPrp17, ScPrp18, ScPrp16 and ScPrp22 can influence 3’ss and alternate BrP site selection [25, 21, 26]. Comparatively, a limited number of studies in *S*. *pombe* correlate splicing factor functions to alternate splice-site choice. Fission yeast factors that promote utilization of off-consensus splice-sites are SpHub1 and *ods1-4* alleles in the genes for U2AF^59^, U2AF^23^, SF1 and Cwf16 splicing factors [23, 27, 28]. SpHub1 promoted splicing of *cdc2+* intron 3 with a sub-optimal 5’ss [23] while the *ods1-4* mutants disrupted the ordered ligation of consecutive exons during constitutive splicing of a multi-intron containing reporter transcript [27, 28]. Recently, *dsk1+*, the fission yeast SR protein kinase, was shown to improve the splicing efficiency of introns with non-consensus 5’ss sequences [29]. Similarly, functional SpPrp4 kinase is needed for the efficient recognition of introns with weak exon1/5’ss interactions and off-consensus branchpoint sequences [30].

Among the well-studied budding yeast second step splicing factors, ScSLU7 is an essential gene that mediates 3’ss choice during second step. ScSLU7 functions also dictate choice between two competing 3’ss as demonstrated by *in vitro* assays [31, 25]. ScPRP18, a non essential factor, strongly interacts with ScSLU7 and bridges its interaction with U5 snRNA during second splicing catalysis step catalysis [32, 33]. Further, both human Slu7 and hPrp18 function during second step in *in vitro* splicing assays [34, 35]. Genome-wide splicing studies from our laboratory utilised the fission yeast missense mutants *spslu7-2* and *spprp18-5* to demonstrate intron-specific functions for these essential factors in the splicing of constitutive exons [36, 37]. In this study, we integrate publicly available fission yeast transcriptome sequences with data from splicing-sensitive microarrays to identify consistent and robust alternative splice events which we then experimentally assessed in fission yeast cells. We demonstrate differing roles for SpSlu7 and SpPrp18 in intron retention/ exon skipping and in the use of alternate splice-sites. Further, environmental stresses affected some splice patterns which we find are differentially dependent on activity of spliceosome factors SpSlu7 and SpPrp18. We also assessed the contribution of intronic *cis* features and the effects of perturbed transcription elongation kinetics on the abundance of some alternative splice isoform choice. Overall, our study sheds more light on the mechanisms by which splice-site recognition and choice is dynamically achieved in the short-intron rich genome of fission yeast which serves as a model for most fungi and for higher eukaryotes like flies, worms and plants.

## Materials and methods

### Yeast strains and plasmid constructions

**Fig 1:**
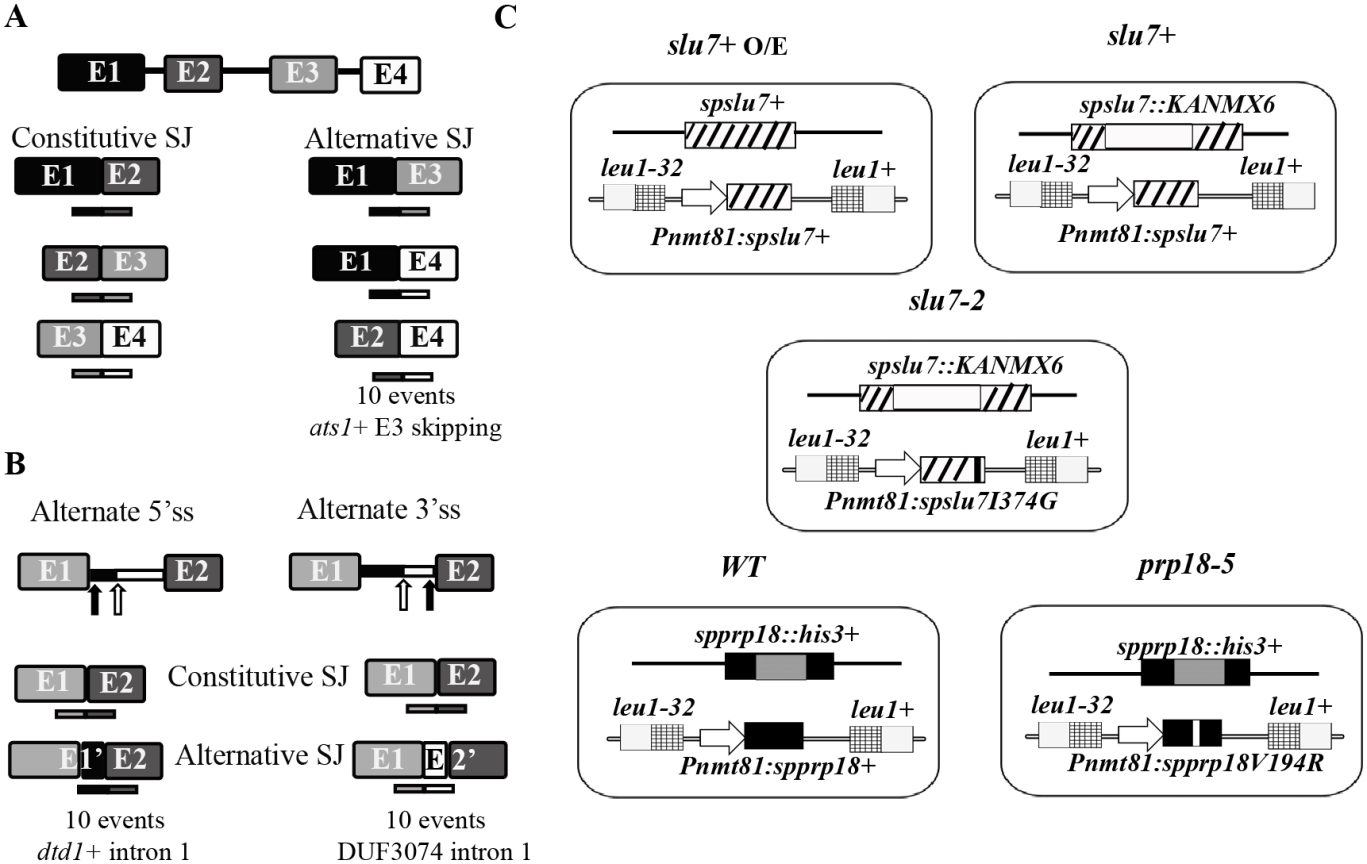
Probe design to detect various *S*. *pombe* alternate splicing events using splicing-sensitive microarray platform. **(A)** Schematic illustration of a representative multi-intronic transcript. Probes on the array for all the theoretically possible splice junctions including constitutive and alternate splice junctions. The number below the schematic of exon-skipped (Alternative SJ) reflects the reproducible events common to different datasets. **(B)** Probes designed to detect mRNA isoforms arising from use of alternative 5’ss (left) or 3’ss (right panel) donor or acceptor sites. Only the 5’ and 3’ exons of the intron with an alternative donor or an acceptor site are schematically represented. Black arrow denotes the canonical /constitutive 5’or 3’ss. White arrow denotes the non-canonical/alternate 5’or 3’ss. The numbers below the schematic for events for alternative donor and acceptor use reflects the reproducible events common to different datasets. (**C**) Diagrammatic illustrations of *slu7*+ O/E, *slu7*+, *slu7-2*, *WT* and *prp18-5* strains having chromosomally integrated wild-type or mutant cDNAs for *slu7*+ and *prp18*+ at the heterologous *leu1*+ locus under thiamine repressible nmt81 promoter.

*S*. *pombe* strains (listed in Table 1) were cultured and analysed as per standard procedures (http://www-rcf.usc.edu/~forsburg, [38]). The wild-type minigene with *ats1*+ (N acetyl transferase) E2-I2-E3-I3-E4 (271 nts) sequences was generated as follows. The indicated gene segments were amplified from wild-type FY528 genomic DNA with the ats1 E2FP and ats1 E4RP primer pair and the PCR product was initially cloned into pBS/KS vector. The gene segment was then excised as EcoRI-XhoI fragment and subcloned into pDBlet vector previously modified with the Ptbp1 promoter. The minigene with ats1 I3 mutation in 5’ss was generated by overlap PCR on the pBS/KS *ats1*+ wild-type minigene template using complementary mutagenic primers and exonic flanking primers (S1 Table) and subsequently subcloned into the pDBlet *Ptbp1* vector. The fission yeast strain *slu7+* O/E (with the wild-type allele under *nmt81* control and the normal complement of the *slu7+* gene); *slu7*+ (with the wild-type allele under *nmt81* control and deletion of endogenous *slu7+* gene), *slu7-2* strain (with missense mutant allele under *nmt81* control and deletion of the endogenous *slu7+* gene) are described in (Fig 1, Table 1). The strains *WT* (with the wild-type allele under *nmt81* control and deletion of endogenous *prp18+* gene) and *prp18-5* (with missense mutant allele under *nmt81* control deletion of endogenous *prp18+* gene) are detailed in [37]. The weak, yet thiamine-repressible, *nmt81* promoter drives the wild-type and variant *prp18+* and *slu7*+ alleles. This promoter was derived from the *nmt1* locus which encodes a thiamine biosynthetic gene, whose transcription is repressed by thiamine supplementation [39]. The strains *slu7+* O/E, *slu7*+ and *slu7-2* were also transformed with the pDBlet *ptbp1 ats1*+ minigene plasmid (*ura4+* marked). All cultures (with or without transformed minigenes) were harvested after 28 hours growth at 30°C in EMM L^-^U^-^ or EMM L^-^ media with or without 15 μM thiamine. The supplementation was done at OD_595_ ~0.02.

**Table 1:** Yeast strains used in this study.

Strain	Genotype	Source
FY527	*h*^-^ *ura4-D18 leu1-32 his3-D1 ade6-M216*	Prof. S. Forsburg
FY528	*h*^+^ *ura4-D18 leu1-32 his3-D1 ade6-M216*	Prof. S. Forsburg
MR3567	*h*^-^ *ura4-D18 leu1-32 upf1*::*KANMX6*	Prof. Paul Russell
*dis3-54*	*h*^-^ *dis3-54 ura4-D18 leu1-32*	Prof. Danesh Moazed
*slu7+* O/E	*h*^-^ *slu7+ leu1*::*pJK148-spslu7ade6 his3-D1 ura4-D18*	Banerjee *et al*., 2013
*slu7+*	*h*^+^*slu7*::*KANMX6 leu1*::*pJK148-spslu7+ ade6 his3-D1 ura4-D18*	This study
*slu7-2*	*h*^+^*slu7*::*KANMX6 leu1*::*pJK148-spslu7 I374G ade6 his3-D1 ura4-D18*	Banerjee *et al*., 2013
*WT*	*h*^-^ *prp18*::*his3*^+^ *leu1*::*pJK148-nmt81 spprp18*^+^ *ade6 his3-D1 ura4-D18*	Vijaykrishna *et al*., 2016
*prp18-5*	*h*^-^ *prp18*::*his3*^+^ *leu1*::*pJK148-nmt81 spprp18-V194R ade6 his3-D1 ura4-D18*	Vijaykrishna *et al*., 2016

Similarly the strains *WT* and *prp18-5* cells with or without the transformed pDBlet *ptbp1 ats1*+ plasmid were harvested after 36 hours growth at 30°C in EMM L^-^U^-^ or EMM L^-^ media again with or without supplementation of 15 μM thiamine which was done at OD_595_ ~0.02. Cell pellets were frozen at -20°C and used further for RNA isolation.

### Probe design to detect alternate splicing events by splicing-sensitive microarrays

#### Exon skipping

In addition to exon-exon probes for every constitutive splice junction (last 25 nts of Exon_n_ + first 25 nts of Exon_n+1_) of *S*. *pombe* exome, splice junction probes (50 nts) were designed for every theoretically possible non-consecutive exon junctions of *S*. *pombe* transcripts (last 25 nts of Exon_x_ + first 25 nts of Exon_y≠x+1_) (Fig 1A).

#### Use of alternate 5’ss as donors

To detect and assess the ten transcripts representing instances of the use of non-canonical 5’ss as reported in [16] by microarrays, additional probes were designed. These probes would hybridise fully to the alternate mRNA isoform arising specifically from use of the non-canonical 5’ss. Use of the non-canonical downstream donor splice site increases the length of the upstream exon (E_n’_). Thus, probes were designed to include last 25 nts of Exon_n’_ that differ from sequences of the constitutive exon, and first 25 nts of Exon_n+1_ downstream exon (Fig 1B). Ten probes were thus designed to capture the use of non-canonical 5’ss using the reported chromosome coordinates [16]. These probes were incorporated in the splicing-sensitive microarray platform used to study the global splicing role for SpPrp18 (probe sequences are enlisted in S2 Table).

#### Use of alternate 3’ss acceptor sites

As described above, data in [16] also reported ten instances of the use of non-canonical acceptor splice sites. Use of the latter results in additional sequences for the downstream exon (Exon_n+1’_) which are absent in the constitutively spliced mRNA (Exonn—Exon_n+1_). Probes, 50 nts in length, were designed to span the last 25 nts of Exon_n_ and 25 nts of new Exon_n+1’_ that can hybridise to the mRNA isoform resulting specifically from use of upstream 3’ss (Fig 1B). These ten specifically designed probes were incorporated to capture these events in the splicing-sensitive microarrays used to study the role of SpPrp18 (probe sequences are enlisted in S2 Table).

#### Datasets used for mapping microarray probe sequences onto transcriptome reads

The MM1 and MM2 transcriptome datasets reported in [40] were from two biological replicates of wild-type fission yeast cells (JB22 972 h^-^) in exponential growth phase in EMM media (MM1 and MM2). The datasets SRX040570 and SRX040571 corresponded to data from wild-type *S*. *pombe* cells (SPY73) grown to mid-log phase in rich YES media [16]. Database encompassing all theoretically possible splice junction probes of 4727 *S*. *pombe* transcripts was generated and then mapped onto the above-mentioned wild-type transcriptome datasets using BOWTIE algorithm. Number of NGS reads mapping to non-consecutive splice junction probes were retrieved from both datasets and the sum total of all reads for a particular probe was determined. 104 exon skipping events had a total read count greater than three that was used as cutoff. The raw intensity values for these probes from splicing microarray data (two replicates) of wild-type *slu7+* O/E strain reported in [36] and in *WT* (*prp18+*) [37] (Fig 1C) were extracted. A high confidence set of top ranking ten exon skipping events was curated based on their higher microarray raw intensity values in *slu7*+ O/E cells.

### Total RNA isolation and reverse transcription of *S*. *pombe* transcripts

Total RNA was isolated using TRI regeant (Sigma) from approximately 20 OD _595_ cell pellet, for all cultures described. 25 μg of total RNA was taken for DNAseI (NEB) treatment and subsequently 2 μg of DNaseI (NEB) treated total RNA was reverse transcribed using MMLV (NEB) and the desired reverse primer. Subsequent PCRs (with the indicated forward and reverse primers) on the cDNAs were done by incorporating αP^32^-dATP for tracer labeling of the amplicons. The separation of the products on native PAGE gel and analyses of the images were done as described in [41]. The primers used for all transcripts analysed are listed in S1 Table. The minitranscript-specific T7 RP was used as the reverse primer in reverse transcription reactions done to assess minitranscripts. The resulting cDNAs were subjected to limiting PCR cycles with the exonic FP and T7 RP.

### Quantitation of the various splice products

Intensities of each of the tracer labelled cDNA amplicons (constitutive, alternative mRNA and unspliced pre-mRNA species) were measured by densitometric analysis (Multigauge) and normalised to the levels of intronless *act1*+ in the same RNA sample. The ratio of normalized alternate mRNA/constitutive mRNA was derived for comparison. Statistical significance for pairwise comparisons between *WT*–T and +T; *prp18-5* –T and +T; *slu7+* -T and *slu7+* +T; *slu7-2* -T and +T was determined using one-way ANOVA with Tukey’s multiple comparison post-test. Data were analyzed using GraphPad Prism v5 (GraphPad Software Inc, CA).

### Culture conditions for growth phase dependent and environmental stress dependent effects

Wild-type fission yeast cells (FY527) were grown in rich media (YES) and cells were harvested at 0.4 OD _595_ (mid-log phase) and OD _595_ 1.5 (stationary phase) as reported in [16, 36]. The harvested cells were immediately frozen at -20°C and RNAs were used to determine the splicing status of candidate transcripts at these growth phases.

The stress treatment regimes followed were as described in [42]. Fission yeast cells were grown to mid-log phase (0.4 OD _595_) at 30°C and shifted to 39°C water bath and cells were harvested at 0, 15 and 30 min for culture aliquots from the start and during the mild heat shock treatment. The cells, in each culture aliquot, were immediately spun down and frozen at -20°C. Similarly, oxidative stress with 1 mM H_2_O_2_ was given for 15 and 60 min to mid-log phase (0.4 OD _595_) cultures, while heavy metal stress was provided to the cells by 0.5 mM CdSO_4_ supplementation for 15 and 30 min.

### Mycophenolic acid treatment

*slu7*+ O/E, *slu7-2*, *WT* and *prp18-5* cells harboring pDBlet *ptbp1 ats1*+, *ats1 I3 mut* or *dtd1* alt. 5’ss mutant minigene plasmids (*ura4+* marked) were cultured in EMM L-U- medium at 30°C until OD _595_ 0.4 was achieved. To these cells, mycophenolic acid (MPA; (Sigma, Cat M5255; 15 μg/mL) was added, incubated for an hour at 30°C and harvested followed by RNA isolation and RT-PCR analysis.

### Western blotting

To detect SpAts1 proteins levels, wild-type cells were transformed with plasmid pREP42HA where full length *ats1+* ORF translationally fused to the HA epitope tag. To assess SpSlu7 protein levels before and after thiamine supplementation (28 hours) western blotting of lysates from *slu7-2* cells was done. Crude whole cell extracts were prepared as reported previously [37]. 30 μl of protein lysate corresponding to 20 μg protein was electrophoresed on SDS-PAGE gels (15% SDS-PAGE for SpAts1 HA; 10% SDS-PAGE for SpSlu7). After electroblotting to Hybond P (GE Healthcare) membranes to detect SpAts1-HA, 1:3500 dilution of mouse monoclonal anti-HA (clone 12CA5) primary antibody (Roche, Cat 11666606001) was used overnight at 4°C. After three 1X TBS (Tris buffered saline) washes 1:5000 dilution of secondary anti-mouse HRP conjugate (Bio-Rad, Cat 1706516) was used for 1 hour at 25°C. To assess SpSlu7 protein levels, 1:3500 dilution of anti-Slu7 rabbit polysera (raised in Vijayraghavan laboratory) was used overnight at 4°C and then 1:10000 dilution of secondary anti-rabbit HRP conjugate (Bio-Rad, Cat 1706518) was used for 1 hour at 25°C. Signals were developed with SuperSignal West Pico Chemiluminescent Substrate (Millipore) and images analysed with Image Quant LAS 4000, GE.

## Results

### Probe design for identification of alternative splice events in fission yeast from splicing-sensitive microarray platform

Previous studies from our laboratory adopted splicing-sensitive microarrays and identified the genome-wide SpSlu7 and SpPrp18 dependent constitutive exon splicing events [36, 37]. Prompted by findings that their budding yeast and human orthologs influence alternative splice-site choice, we aimed to investigate here roles for these fission yeast factors in alternatively spliced or exon skipped isoforms in our fission yeast splicing-sensitive microarray data. We first deduced alternative splice events that occur in wild-type cells and are reproducibly detected in multiple experimental platforms. A primary database of 9452 probes sequences for every conceivable splice junction in the genome’s 4727 *S*. *pombe* transcripts were incorporated on the microarrays. Of these 9452 splice junction probes, 4603 and 4849 probes were for consecutive exon-exon (consecutive SJ) and non-consecutive junctions (alternative SJ), respectively (Fig 1A and S1A Fig). These compiled splice junction sequences were mapped onto two independently reported transcriptomes of exponentially growing wild-type fission yeast cultures [40, 16]. This analysis identified only 104 non-consecutive (exon skipped) splice events as common to both reported transcriptome datasets and we inferred these as stringent and reproducible events (S3 Table). Most of these events were of very low abundance, only 17 exon skipped junctions had total next generation sequence (NGS) read count more than 10 reaffirming that in wild-type cells exon skipping, even when it occurs is at low levels. To deduce any role for the splicing factor SpSlu7 in these events, we first interrogated our microarray data [36] from two biological replicates of stationary phase *slu7*+ O/E cells (expressed from native locus and the integrated copy at the *leu1* locus; Fig 1C) to identify exon skipped events detected by the custom microarray probes. As around 74 events detected by very deep NGS transcriptome studies were also captured in both our microarray data, we highlight the sensitivity, robustness and suitability of splicing-sensitive microarrays to study even low abundance splice isoforms. By these combined approaches and careful scrutiny we curated ten high-confidence exon skipping events (listed in Table 2) that are detected reliabily across varied experimental platforms. Skipped exons in higher eukaryotes are reported to be short and their downstream introns generally have weaker 5’ss sequences [43]. We note that the 5’ss sequences of fission yeast introns downstream of most of these ten skipped exons often (7/10 cases) are non-consensus. The *ats1+* (SPAC1002.07c) with the exon 3 skipped isoform and a downstream intron with non-consensus 5’ss GUAGUG was taken as a representative for this class of alternative splicing events (Table 2) for further experimental studies.

**Table 2:** Ten high-confidence exon skipping events in fission yeast.

Gene Name	Alternate Exon junction	RNA seq reads	Skipped exon length (nts)	5’ss of downstream intron
40S ribosomal protein S30	E1-E3	41	129	GUAAGU
40S ribosomal protein S13	E1-E3	36	33	GUAUGC
DUF757 family protein	E1-E3	17	12	GUACGU
translocon beta subunit Sbh1	E1-E3	13	62	GUUCGU
ADP-ribosylation factor Alp41	E2-E4	11	73	GUACGU
N-acetyltransferase Ats1	E2-E4	10	35	GUAGUG
ubiquinol-cytochrome-c reductase complex subunit 7	E1-E3	9	21	GUAAGU
1-(5- phosphoribosyl)-5-[(5-phosphoribosylamino) methylideneamino]imidazole-4-carboxamide isomerase	E1-E3	8	71	GUAAAU
ubiquinol-cytochrome-c reductase complex subunit 8	E1-E4	7	72, 189	GUUCGC
ubiquinol-cytochrome-c reductase complex subunit 8	E1-E3	7	72	GUAAGU

Underlined are the instances where the 5’ss of the intron downstream to the skipped exon deviate from consensus or where skipped exon lengths are smaller than 35 nts.

We also assessed the prevalence of other splice isoforms where alternate 5’ or 3’ ss for a given exon-intron junction were used. Comparative analysis of deep transcriptomes in *S*. *pombe*, *S*. *octosporus*, *S*. *cryophilus* and *S*. *japonicus* genomes [16] had identified a small subset of transcripts where such 5’ or 3’ alternative splice sites in an intron were used apart from the bonafide splice sites. Based on the chromosome coordinates of these few *S*. *pombe* transcripts we had incorporated custom designed probes (Fig 1B and S1B Fig) in a more recent splicing microarray study from our laboratory [37] where the SpPrp18 global functions were addressed [37]. Despite the inherent disadvantage of splicing microarrays not being as robust as deep transcriptome sequencing we surprisingly found custom microarrays are sensitive enough to capture some alternate splicing events in cellular transcripts (Table 3). We observed the suboptimal 5’ss (GUAAUA) in the intron 1 of *dtd1*+ (D-tyr-tRNA deacylase; SPAC8C9.05) to be a reproducible example for such an alternative mRNA form in *WT* RNA samples (Table 3). Similarly, microarray data revealed the use of a non-canonical 3’ss in intron 1 of the transcript for DUF3074 family protein (SPBC577.11) (Table 3). Overall, we present a facile strategy to utilise publicly available datasets to reliably detect alternative isoforms by microarrays and thus identified candidate alternative splice events for experimental studies on roles for spliceosomal factors and the effects of environmental stresses.

**Table 3:** Microarray signal raw intensities for custom designed probes to detect use of alternative use of splice sites in two biological replicates of wild-type (*WT*-T; *prp18*+) cells.

Alternative donor candidates [16]	Microarray signal intensity in WT-T for custom designed probes		Alternative acceptor candidates [16]	Microarray signal intensity in WT-T for custom designed probes	
	#1	#2		#1	#2
asmbl_14785_donor	2.2	3.1	asmbl_14785_accept	2.2	3.1
asmbl_16630_donor	2.1	21.1	asmbl_18393_accept	5.0	4.6
asmbl_2356_donor	123.3	1135.2	asmbl_3133_accept	41.0	86.5
asmbl_5336_donor	2.2	3.1	asmbl_7672_accept	3.5	3.1
asmbl_8229_donor	2.3	3.1	SPAC17C9.11c	11.4	27.7
*toe1*+	2.2	3.1	*ppk9*+	3.2	3.6
*dtd1*+	49.2	87.3	SPBC3E7.04c	2.2	3.1
*prp38*+	41.2	62.7	DUF3074 family protein	2212.7	4551.4
*btb2*+	2.3	3.2	*wtf11*+	161.7	1334.3
*vma8*+	8.4	16.8	*hnt3*+	2.5	3.4

### *In vivo* validation of the alternative splicing events in wild-type cells during log and stationary phase

**Fig 2:**
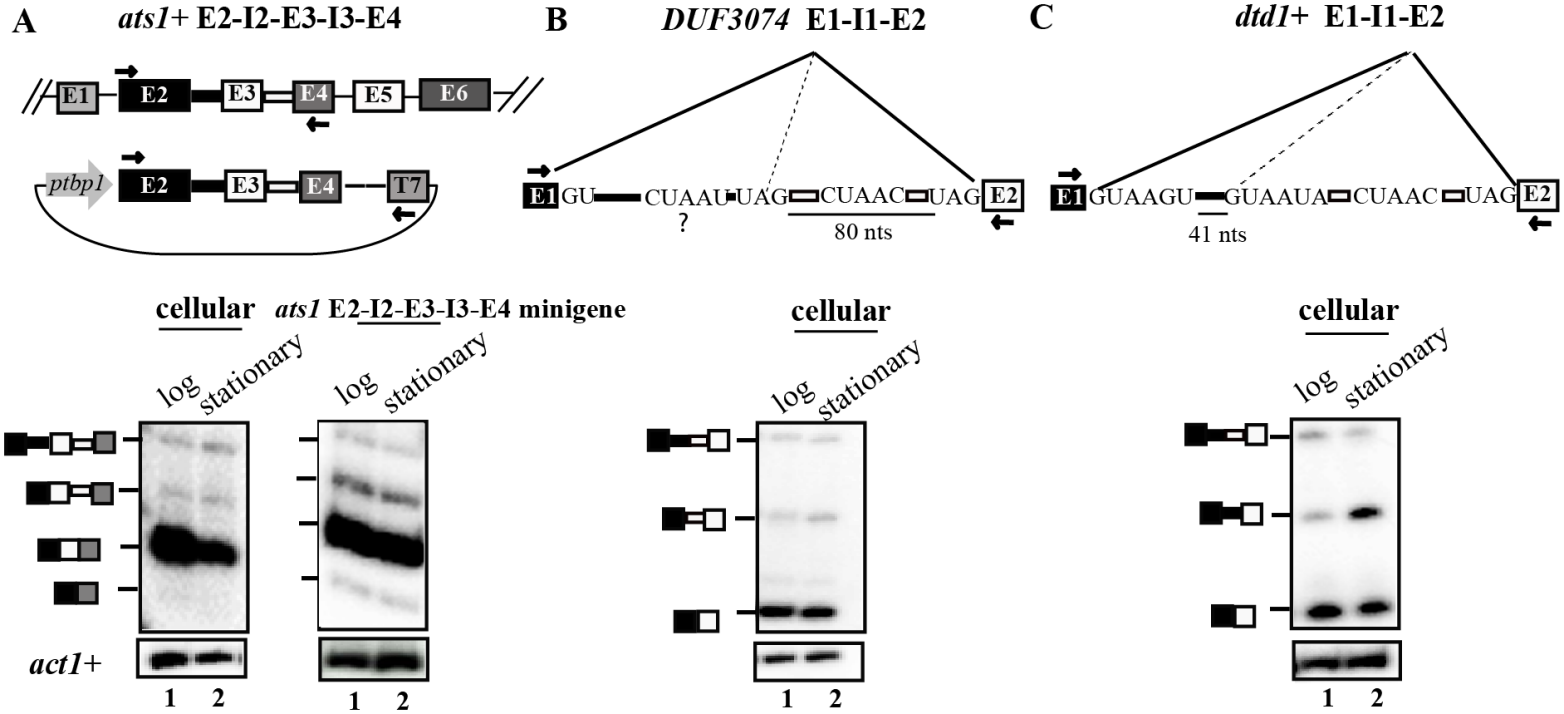
Validation of alternative splice events in wild-type *S*. *pombe* cultures at different growth phases. **(A)** Schematic representation of cellular *ats1*+ transcript and the plasmid expressed minitranscript *ats1*+ E2-I2-E3-I3-E4 transcribed from the *tbp1* promoter. Arrows indicate primers used for reverse transcription (RT) and PCR. Semi-quantitiative RT-PCR, tracer-labelled with αP^32^-dATP and performed with E2 FP-E4 RP primer pair, detects cellular *ats1*+ transcripts in wild-type cells (gel image in the left panel) while RT-PCR with E2 FP and T7 primer selectively detects plasmid expressed minitranscript only (gel image in the right panel). The data for wild-type cells in log and stationary phase are shown. Intronless *act1*+ served as the normalising control. **(B)** Schematic of cellular DUF3074 E1-I1-E2 RNA with the canonical and non-canonical 3’ss sequences along with the 5’ss, canonical BrP and second predicted BrP denoted by “?”. **(C)** Diagrammatic representation of E1-I1-E2 in the *dtd1*+ transcript depicting the canonical and non-canonical 5’ss sequence, the distance between these donor sites and the position of the BrP and 3’ss. Semi-quantitative RT-PCR analyses done with flanking exonic primers detected the constitutive and alternatively spliced products for DUF3074 and *dtd1*+ transcripts in wild-type cells during log and stationary phase. All PCR products were separated on 8% native PAGE gels. The identity of the splice isoforms are schematically shown to the left of the corresponding gel images.

Next, we experimentally validated few alternative splicing events common to transcriptome sequencing and splicing microarray datasets by semi-quantitative RT-PCR (Fig 1; [16, 36, 37]. Wild-type *S*. *pombe* cells were grown to mid-log and stationary phase, as described in materials and methods, and the cellular splice isoforms from *ats1*+, DUF3074 and *dtd1+* transcripts were examined by RT-PCR assays. In each case, oligos complementary to exons flanking the skipped exon/alternate splice sites to be tested were used to simultaneously assess both constitutive and alternative splicing. As expected from microarray raw data and transcriptome reads for *ats1*+, we observed abundant levels of fully spliced mRNA (E2-E3-E4, constitutive SJ), basal levels of unspliced pre-mRNA (E2-I2-E3-I3-E4), and very low levels of E3 skipped mRNA (Fig 2A, gel image in left panel). Additionally in these wild-type cells, we consistently detected an additional splice isoform migrating between the unspliced pre-mRNA and spliced mRNA (Fig 2A, gel image in left panel) that we identified as an intron 3 (I3) retained mRNA through sequencing (S2C Fig). The I3 retained mRNA was at considerable lower abundance in comparison to the fully spliced mRNA. In order facilitate the detection of these low abundance splice isoforms, we generated a minigene comprising *ats1*+ E2-I2-E3-I3-E4 gene segments in the fission yeast shuttle vector pDBlet for expression of a minitranscript from the heterologous *tbp1* promoter (Fig 2A, schematic). The splicing profile observed for *ats1*+ E2-I2-E3-I3-E4 in cellular transcripts (Fig 2A, gel image in left panel) were recapitulated in the minitranscript and it facilitated detection of I3 retained mRNA and E3 skipped mRNA isoforms (Fig 2A, gel image in right panel). The splicing profile for *ats1*+ E2-I2-E3-I3-E4 minitranscript did not vary between cultures at log *versus* stationary phase of growth. Next, the splicing profile for DUF3074 intron 1 was assayed by RT-PCR to determine whether utilisation of the alternative 3’ss located 80 nts upstream of canonical 3’ss produced an alternative mRNA isoform. A predicted BrP sequence (CUAAU) is positioned 13 nts upstream of this alternative 3’ss as shown in the schematic (Fig 2B). In wild-type cells the fully spliced constitutive mRNA and basal levels of unspliced pre-mRNA (E1-I1-E2) were detected at similar levels in cells at log and stationary phases of growth (Fig 2B). We validated by RT-PCR the presence of low levels of alternative mRNA for the *dtd1*+ transcript arising from the use of the sub-optimal 5’ss (GUAAUA) located 41 nts downstream to the canonical 5’ss (GUAAGU) of *dtd1*+ intron 1. Interestingly, a 3-fold increase in this alternative *dtd1*+ mRNA isoform occurred in the cells at the stationary phase as compared to log phase cultures (Fig 2C, lanes 1 and 2) indicating a growth-stage specific change in utilisation of 5’ splice sites of this intron.

### Roles for SpSlu7 and SpPrp18 in *ats1*+ intron 3 retention, exon 3 skipping and their relationship to splice site elements

**Fig 3:**
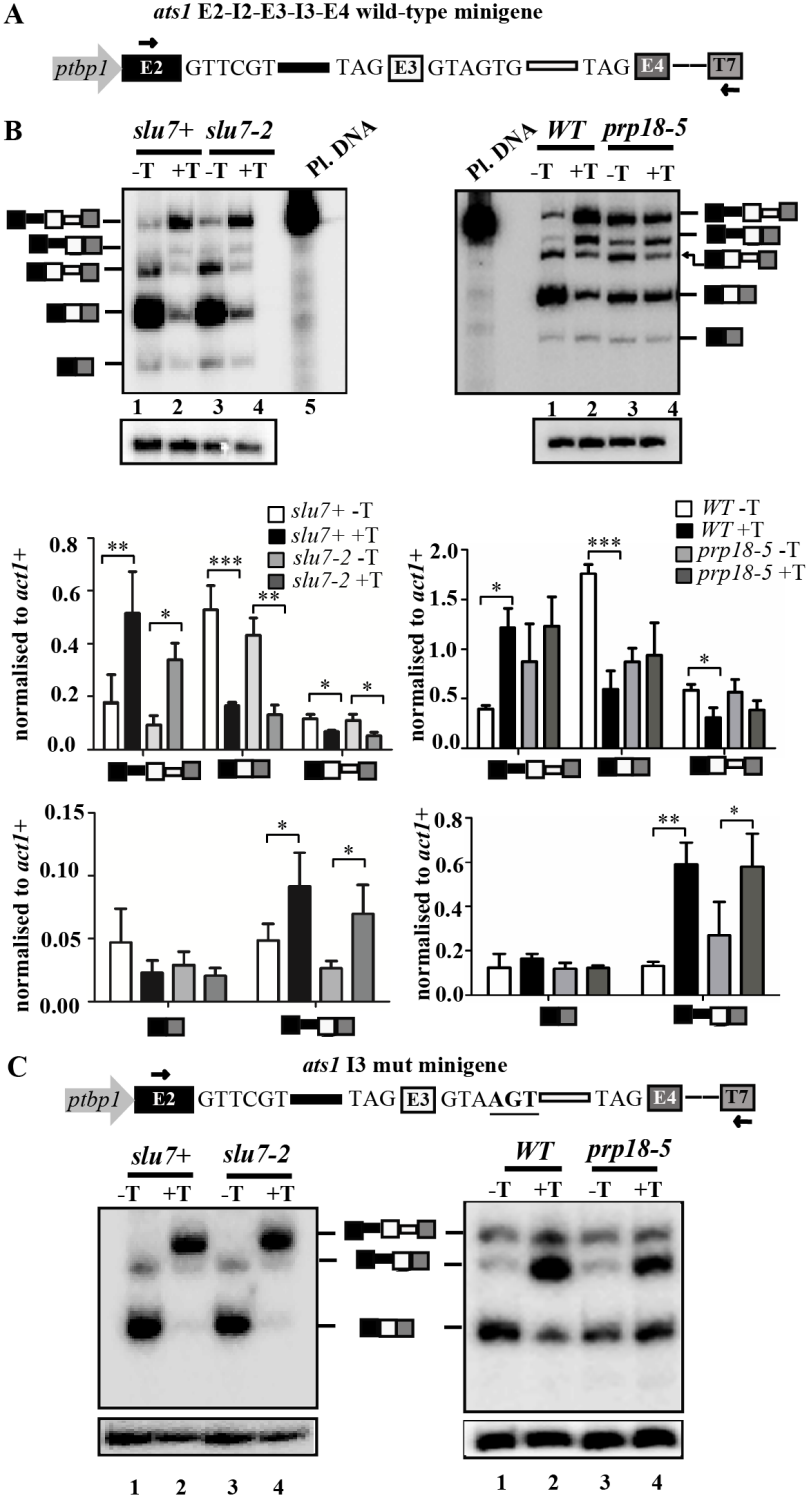
SpSplu7 and SpPrp18 influence *ats1*+ constitutive and alternative splicing. **(A)** Exon intron architecture of the plasmid minigene *ats1*+ E2-I2-E3-I3-E4 to express wild-type minitranscripts from the *tbp1* promoter. **(B)** RT-PCR with the T7 reverse primer specific to the minitranscript and the exon 2 forward primer was performed on RNA from *slu7*+, *slu7-2*, *WT* and *prp18-5* strains all of which were transformed with the *ats1*+ wild-type minigene. All cultures were grown in the presence (+T) or in the absence (-T) of 15 μM thiamine. After densitometric analysis of the levels of various spliced isoforms they were normalised to intronless *act1*+ transcript and data was plotted as bar graphs. Statistical significance for the various comparisons between *WT*–T and +T; *prp18-5* –T and +T; *slu7+* -T and *slu7+* +T; *slu7-2* -T and +T was determined using one-way ANOVA with Tukey’s multiple comparison post-test; *p < 0.05, **p < 0.01, ***p < 0.001, ns: non-significant.The identity of the various mRNA isoforms is schematically indicated adjacent to the gel images. **(C)** Schematic representation of the mutant minigene *ats1* I3 mut where mutations in the non-canonical 5’ss are shown. This plasmid was transformed in *slu7*+, *slu7-2*, *WT* and *prp18-5* strains. Comparative RT-PCR analysis of the splicing status of *ats1* mutant minitranscript in all cultures grown in the presence (+T) and absence (-T) of 15 μM thiamine (left and right panels).

Our prior studies used strains with conditionally expressed missense mutants in the splicing factors SpSlu7 and SpPrp18 and splicing-sensitive microarray to infer that these essential factors have intron-specific functions for constitutive splicing (Table 1; Fig 1C) [36, 37]. In these microarray data, raw intensities for majority of the ten high confidence exon skipping events described above (Table 2) were largely unaltered when levels of functional SpSlu7 and SpPrp18 were depleted i.e., in thiamine treated *slu7-2* +T and *WT* +T cultures. However, marginally reduced array signal for *ats1+* exon 3 skipped form was seen in wild-type Slu7 expressing cells and also when Slu7-2 missense mutant was metabolically depleted (*slu7-2* +T). Such changes in array signal intensities were interestingly not seen upon depletion of SpPrp18. To validate these microarray data we examined the *in vivo* constitutive and alternatively spliced isoforms for the plasmid expressed minitranscript *ats1*+ E2-I2-E3-I3-E4 (Fig 3A). These were studied in the strains *slu7+* and *slu7*-2 (Fig 1C) grown under conditions that express or metabolically deplete the wild-type Slu7 or Slu7-2 missense protein (S3A Fig). RT-PCR analyses revealed a clear dependence on SpSlu7 for the constitutive splicing of *ats1*+ intron 2 and 3 as a 3-fold increase in the levels of unspliced pre-mRNA (E2-I2-E3-I3-E4) and similar level of decrease in the constitutive spliced mRNA levels (E2-E3-E4) occur upon metabolic depletion of this splicing factor (Fig 3B, left panel, lanes 2 and 4). These RT-PCR analyses indicated that the intron 3 retained *ats1+* splice isoform occurs at 3-fold lower levels than the completely spliced constitutive mRNA isoform even in cells with functional Slu7 (Fig 3B, left panel, lane 1). These intron 3 retained isoform levels are further reduced on depletion of the wild type Slu7 or missense protein Slu7-2 (Fig 3B, left panel, lanes 2 and 4). Thus these findings hint at a role for SpSlu7 in the retention of *ats1*+ intron 3. Furthermore, on thiamine supplementation, we observed the accumulation of an additional cDNA species shorter than the unspliced pre-mRNA species. This cDNA was sequenced to establish that it corresponded to the intron 2 (I2) retained isoform (E2-I2-E3-E4) wherein intron 3 was spliced out (S2C Fig). These findings indicate that the levels of I2 and I3 retained mRNAs might have an opposing expression pattern. We also performed similar RT-PCR analyses for *ats1+* minitranscript splicing in *prp18+* and *prp18-5* cells (Fig 1C). The metabolic depletion of wild-type SpPrp18 in the *WT* strain [37] resulted in a significant arrest of constitutive splicing as manifested by unspliced pre-mRNA (E2-I2-E3-I3-E4) accumulation and decrease of constitutively spliced (E2-E3-E4) mRNA levels (Fig 3B, right panel, lane 2). The levels of unspliced pre-mRNA (E2-I2-E3-I3-E4) were elevated in *prp18-5* cells even when the mutant protein was expressed (Fig 3B, right panel, lane 3) thereby supporting the notion that the splicing of *ats1+* intron 2 and intron 3 are dependent on fully functional SpPrp18. Thiamine-based depletion of *prp18-5* mutant protein [32] did not further exacerbate the splicing defects of *ats1*+ (Fig 3B, right panel, lanes 3 and 4). The I3 retained splice isoform levels were also reduced upon depletion of SpPrp18 (Fig 3B, right panel, lanes 2, 4); thus retention of intron 3 was lower in absence of either SpPrp18 or SpSlu7. Notably, depletion of either SpPrp18 of SpPrp18-5 caused greater degree of intron 2 retention (Fig 3B, right panel) as compared to milder effects on depletion of Slu7 (Fig 3B, left panel, lane 4). The levels of low abundance *ats1*+ E3 skipped isoform were marginally reduced in cells treated to deplete SpSlu7 or SpSlu7-2 protein (Fig 3A; left panel, lanes 2 and 4) while the absence of SpPrp18 did not alter levels of this isoform.

Interestingly, both intron 2 and 3 of *ats1+*, that are variably subjected to retention, harbour non-consensus 5’ss sequences, GUUCGU and GUAGUG respectively which deviate from the consensus GUAA/UGU hexamer motif. Sub-optimal 5’ ss can result in intron retention as well as skipping of upstream exon [42] (Table 2). We therefore mutagenized the *ats1*+ I3 5’ ss motif GTAGTG, in the *ats1* I3 mut minigene plasmid construct, to thereby transcribe a minitranscript with an optimal 5’ ss GUAAGU sequence (Fig 3C, schematic). We then assessed splicing efficiency in RNA from *slu7*+ cells transformed with the *ats1*+ intron 3 mutant plasmid. We observed in cells with Slu7 protein the I3 retained mRNA isoform as well as the E3 skipped mRNA isoform were entirely absent (Fig 3C, left panel, lanes 1, 3). These data demonstrate that the weak 5’ss contributed to the retention of intron 3 of *ats1*+ in wild-type cells. Metabolic depletion of Slu7 or Slu7-2 had strong effects on constitutive splicing as unspliced pre-mRNA accumulated and constitutively spliced mRNA levels are reduced for the minitranscript with mutant 5’ss (Fig 3C, left panel, lanes 2, 4). The levels of I2 retained mRNA isoform levels were also lowered. The effects of these mutations in the 5’ss of *ats1* I3 minitranscripts were also tested in *WT* and *prp18-5* cells. These RT-PCRs showed I3 retained and E3 skipped isoforms were again absent while there was a strong increase in I2 retained mRNA isoform when cells are depleted of either wild-type and mutant SpPrp18 (Fig 3C, right panel, lanes 2 and 4). These data showed that creating a consensus 5’ss in intron 3 possibly stalled the splicing of intron 2 while intron 3 is possibly was spliced to similar efficiencies in *WT* and *prp18-5* cells (Fig 3C, right panel, lanes 2 and 4). These observations also indicate the opposing expression levels of I2 and I3 intron retention events. We reason that the increased retention of *ats1*+ intron 2, when the non-consensus 5’splice-site in intron 3 is optimized, is plausibly driven by other elements in intron 2. Our data show that SpPrp18 has a greater role in the splicing of *ats1*+ intron 2 as compared to SpSlu7. Together, these data show that alternative splicing events in a multi-intronic transcript (*ats1*+) are in some instances dependent on both these splicing factors yet other cases have differing dependencies that cumulatively account for the observed ratios of various splice isoforms in wild-type cells.

### Differential influence of SpSlu7 and SpPrp18 on the use of alternative 5’ and 3’ splice sites

**Fig 4:**
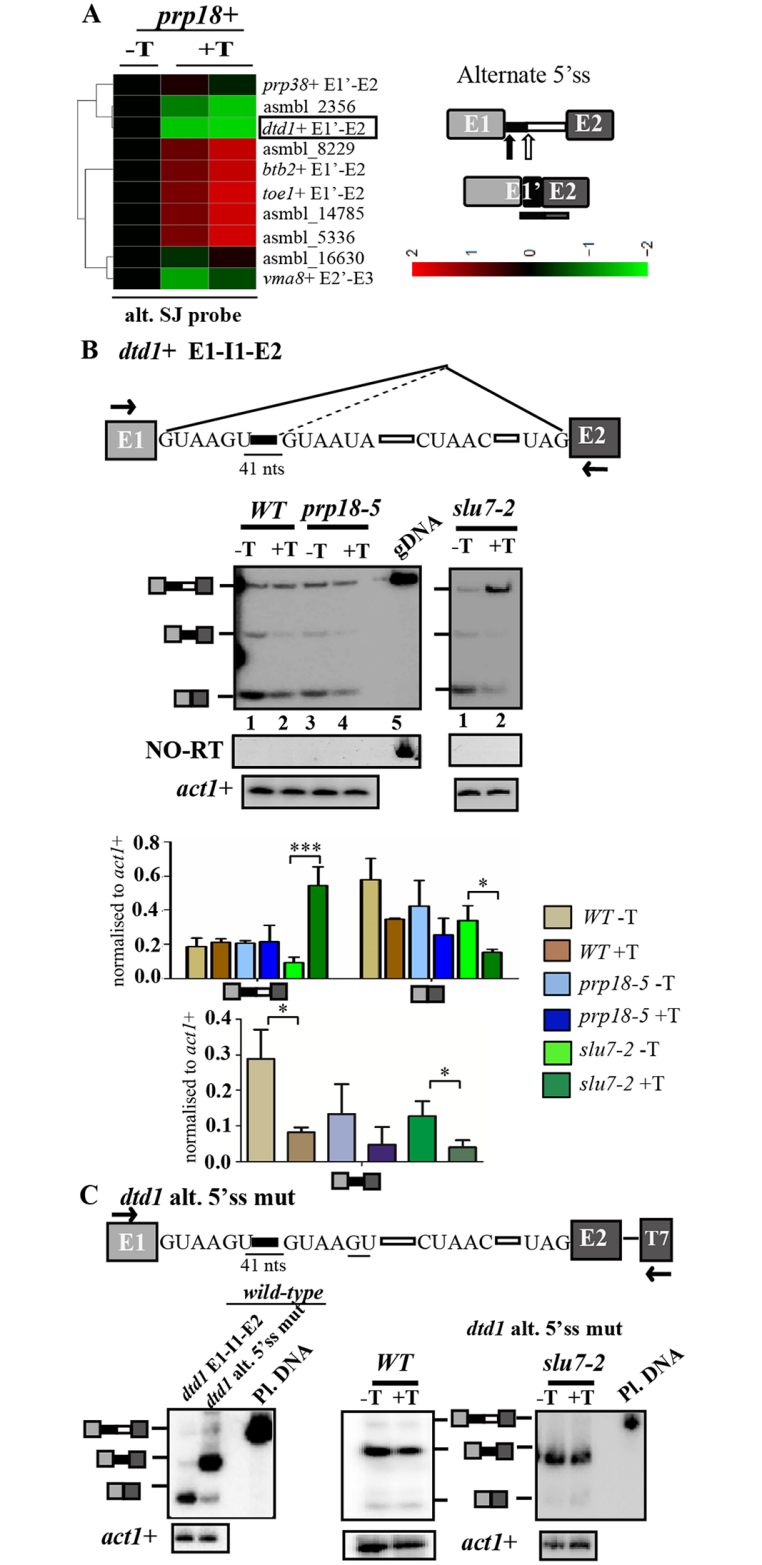
Differential utilisation of downstream non-canonical 5’ss of *dtd1*+ intron 1 by SpSlu7 and SpPrp18. **(A)** Heatmap representing the fold changes in raw intensity values for ten probes for alternate mRNA arising from use of alternative 5’ss in RNA from *WT*+T cells (thiamine supplemented) after normalization to intensities in *WT*-T cells. Data from two biological replicates for the treatment regime were given. The complementarity of these probes to the alternative mRNA is shown to the right. **(B)** Diagrammatic representation of E1-I1-E2 in *dtd1*+ cellular RNA depicting the canonical and non-canonical 5’ss sequences along with the BrP and 3’ss. The distance between the canonical and non-canonical 5’ss is given. RT-PCR assays using primers positioned in exon 1 and exon 2 to simultanenously detect the constitutive and alternate mRNA isoforms in *WT* and *prp18-5* cultures grown in the presence or absence of thiamine (-T and +T). Similar analysis was performed for *slu7-2* cultures grown in the presence or in the absence of thiamine. PCR on genomic DNA (gDNA) template served as a marker for migration position of unspliced pre-mRNA. NO-RT PCR was carried out directly on the RNA samples using the same set of flanking exonic primers. Intronless *act1*+ transcript served as the normalising control. After densitometric analysis of the various spliced products, the normalised levels of pre-mRNA, constitutive mRNA and alternate mRNA were plotted as bar graphs and the ratio of alternate mRNA to constitutive mRNA was plotted. Statistical significance for the various comparisons between *WT*–T and +T; *prp18-5* –T and +T; *slu7*+ -T and +T and *slu7-2* -T and +T was determined using one-way ANOVA with Tukey’s multiple comparison post-test; *p < 0.05, **p < 0.01, ***p < 0.001, ns: non-significant. **(C)** Schematic illustration of plasmid expressed minitranscript where the *dtd1* alt 5’ss is mutated. The mutations in non-canonical 5’ss are underlined. Left panel, comparative RT-PCR study of the splicing status for *dtd1* alt 5’ss mutant minitranscript and the wild-type *dtd1*+ E1-I1-E2 minitranscript in RNA from wild-type cells. The primers used in RT-PCR were *dtd1*+ E1FP and T7 primers. Shown to the right are RT-PCR analyses of RNA from *WT* (*prp18+*) and *slu7-2* cells transformed with plasmid minigene *dtd1* alt 5’ss mutant. Both cultures were grown in the absence and presence of thiamine (-T and +T). Intronless *act1*+ served as normalising control.

Data from splicing sensitive microarrays were used to derive normalized raw intensity signals from custom designed probes that reflect use of alternative 5’ss elements. Specifically, the signal intensities in two replicates of RNA from *WT* +T (i.e. *prp18+* depleted) cells were normalised to those in the *WT*-T untreated sample (i.e., expressing *prp18+*). The fold changes for some instances of altered 5’ss use are represented as a heatmap (Fig 4A). The use of downstream non-canonical 5’ss in transcripts such as *prp38+*, *vma8+* and *dtd1*+ are shown (Fig 4A, Table 3). We chose the cellular *dtd1*+ transcript for further investigation by RT-PCR assays as in microarrays we noted a statistically significant and reproducibly reduced signal for its alternate mRNA isoform (Fig 4A, marked in black box). In accordance with the microarray results, the RT-PCR assay also showed reduced levels of the alternate isoform on depletion of the SpPrp18 wild-type as well as the missense protein (Fig 4B, left panel, lanes 2 and 4). With regard to the constitutive splicing of *dtd1*+ intron 1 these experiments show that this intron, unlike other cellular introns (S3B Fig), is only marginally dependent on functional SpPrp18 as its depletion only affected constitutive mRNA (E1-E2) levels without an increase in unspliced pre-mRNA. These data suggest that constitutive splicing of *dtd1*+ intron 1 is not strongly dependent on SpPrp18. However, we do not rule out the possibility that the reduced levels of the *dtd1*+ constitutive mRNA (E1-E2) could be due to the indirect effects of thiamine-based SpPrp18 metabolic depletion. These results hint at additional new roles for SpPrp18 in the utilization of sub-optimal 5’ss in certain introns.

We also investigated the role of SpSlu7 in the use of the two 5’ss in *dtd1*+ I1 by employing RNA from strains expressing or those depleted of Slu7-2 mutant protein. A clear dependence on SpSlu7 for the constitive splicing of *dtd1*+ I1 was reflected by the nearly 5-fold increase in unspliced pre-mRNA (E1-I1-E2) and concomitant 2-fold decrease in constitutively spliced E1-E2 mRNA levels (Fig 4B, right panel, lanes 1 and 2). Low levels of the alternate mRNA (E1’-E2), arising from the downstream 5’ss, were also detected in cells with Slu7-2 protein (i.e., *slu7-2* -T). This alternative mRNA isoform level decreased on depletion of mutant Slu7-2 protein (Fig 4B, gel image in right panel lanes 1, 2). Thus, SpSlu7 was required for the use of both the canonical and non-canonical 5’ss in the *dtd1+* intron 1, while SpPrp18 activity was required for optimal basal level usage of the non-canonical 5’ss.

To test whether the non-consensus nature of the *dtd1*+ I1 5’ss (GUAAUA) contributed to the low levels of the alternate mRNA, we mutagenized this 5’ss to fit the global consensus (GUAAGU). The mutant minigene termed *dtd1* alt 5’ss mut E1-I1-E2 (Fig 4C, schematic) was transformed into the wild-type cells and tested for splicing efficiency. We observed a dramatic increase in alternate mRNA to levels higher than the normal constitutively mRNA formed by use of the wild-type optimal 5’ss (Fig 4C, left panel). This indicated that the weak consensus of the alternative 5’ss was indeed responsible for its low level utilization in wild-type cells. Interestingly, the increased utilization of the alternate 5’ss occurred even in cells depleted of SpPrp18 or Slu7-2 missense protein (Fig 4C, middle and right panels). The variant intron with the alternate 5’ss could thus be spliced independent of SpPrp18 or SpSlu7. This was unlike the wild-type minitranscript, wherein there is a predominant use of the canonical 5’ss and minor usage of alternative 5’ss.

**Fig 5:**
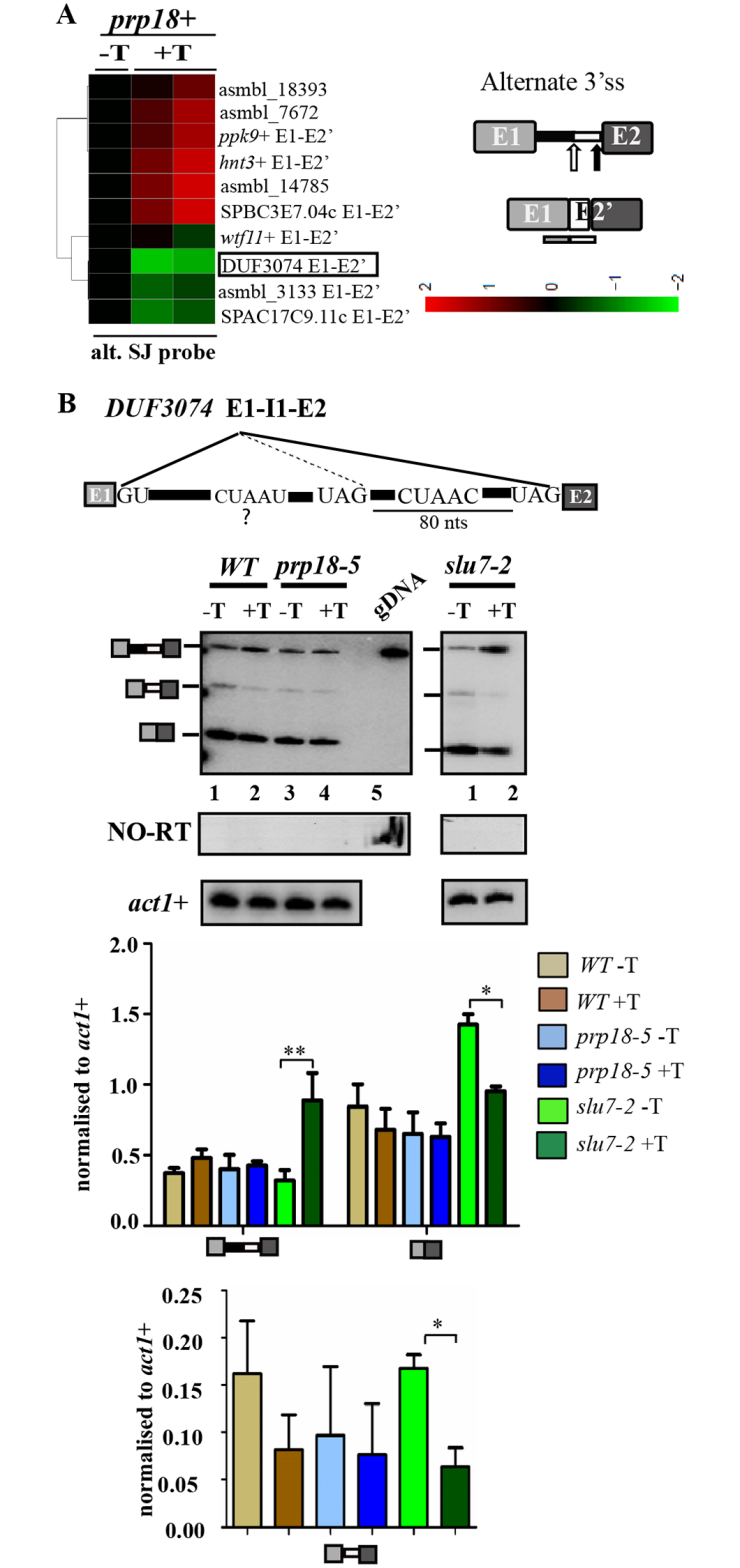
Utilisation of the non-canonical 3’ss in DUF3074 intron 1. **(A)** Heatmap representing the fold changes in microarray raw intensity values for ten specific probes that detect alternative mRNAs produced by the use of alternative 3’ss. The probe complementarity to the alternative mRNA is shown to the right. Array data is presented for two biological replicates of RNA from *WT* +T cells (thiamine treated; *prp18*+ depleted) normalised to *WT*-T cells (untreated). **(B)** Illustration of E1-I1-E2 in the DUF3074 transcript with the canonical and alternative 3’ss sequences, the distance between these sequences. The 5’ss, canonical BrP element and a predicted BrP upstream to alternative 3’ss are shown. RT-PCR assays using primers for the flanking exons in DUF3074 intron 1 to simultaneously detect amplicons from the unspliced pre-mRNA, constitutive mRNA and the alternate mRNA produced from use of the non-canonical 3’ss. The analyses were performed in RNA from *WT*, *prp18-5* and *slu7-2* cultures grown in the presence or absence of 15 μM thiamine. NO-RT PCR was carried out directly on the RNA samples using the same set of flanking exonic primers. The normalised values for unspliced and spliced mRNA isoforms are plotted as bar graphs along with the ratio for alternate mRNA to constitutive mRNA forms. Statistical significance for the comparison between *slu7-2* -T and +T was determined using one-way ANOVA with Tukey’s multiple comparison post-test; *p < 0.05, **p < 0.01, ***p < 0.001, ns: non-significant.

Budding yeast ScSlu7 and ScPrp18 in *in vitro* splicing assays, influence 3’ss use in modified *ACT1* intron containing transcripts [44, 45]. Here, using our fission yeast microarray data we first compared the fold changes in the raw intensity values for probes reporting use of alternative 3’ss and thereby identified mRNAs with new splice junctions. Candidates transcripts with such altered mRNAs included *wtf11*+, DUF3074 family protein and SPAC17C9.11c (Fig 5A). Semi-quantitative RT-PCR analyses for splicing of DUF3074 intron 1 (Fig 5B, lane 1, 2) were concordant with the statistically significant data from microarrays (highlighted in black box, Fig 5A). Use of the canonical or the non-canonical 3’ss in DUF3074 I1 is only very marginally dependent on SpPrp18 for constitutive or alternative splicing as there is only a subtle change in these mRNA levels on depletion of SpPrp18 (Fig 5B, left panel, lanes 2–4). In contrast, similar RT-PCR assays on DUF3074 intron 1 in RNA from cells depleted of Slu7-2 (*slu7-2* +T) clearly show that the absence of functional SpSlu7 affects use of the canonical 3’ss as the constitutively spliced mRNA levels were low (Fig 5B, right panel, lanes 1 and 2). Moreover, the levels of alternate mRNA isoform were also significantly decreased in *slu7-2* +T samples where splicing factor was depleted. These data imply SpSlu7 influences the utilisation of both canonical and non-canonical 3’ss in the DUF3074 intron 1.

### Assessment of the biological significance for alternatively spliced events in fission yeast

In fission yeast, as also in budding yeast, alternatively spliced RNAs occur at higher levels in RNA surveillance mutants indicating a possible regulatory function in fine tuning steady state transcript levels rather than in increasing the proteome diversity [19, 21]. The alternative mRNA isoforms for *ats1*+, DUF3074 and *dtd1*+ studied here all bear premature termination codons (PTCs) and hence are unlikely to form protein isoforms. We analysed the relative abundance of splice isoforms for these transcripts in various RNA surveillance pathway mutants. A compromise in the NMD machinery was achieved by using the *upf1Δ* strain [46] while partial inactivation of exosome mediated RNA degradation was obtained using the *dis3-54* mutant [47, 48]. We found no increase in the levels of *ats1+* I3 retained or E3 skipped mRNA isoforms in these RNA surveillance mutants (S4A Fig, lanes 1–3). In the *dis3-54* exosome mutant we observed a marginal increase in unspliced pre-mRNA to mRNA ratio (S4A Fig, lane 3) as similarly previously reported for selected transcripts [49]. To examine the possibility of novel protein isoforms from the *ats1*+ alternatively spliced RNAs, we generated a plasmid construct to express N-terminal HA epitope-tagged full length Ats1 protein and its variants, if any. Immunoblotting with anti-HA antibody readily detected the 21 kDa full length protein (S4D Fig) but other protein isoforms i.e., a 11 kDa protein from I3 retained mRNA or a 12 kDa protein from E3 skipped mRNA splice isoforms were not detected.

The levels of alternative splice isoform mRNAs for *dtd1*+ and DUF3074 were also not much stabilized in the genetic background of *upf1*Δ and *dis3-54* mutant (S4B and S4C Fig). These observations suggest that the alternative spliced isoforms of *ats1+*, *dtd1+* and DUF3074 are perhaps not direct substrates for the two RNA surveillance mechanisms tested here.

### Stress-specific regulation of alternative splicing

**Fig 6:**
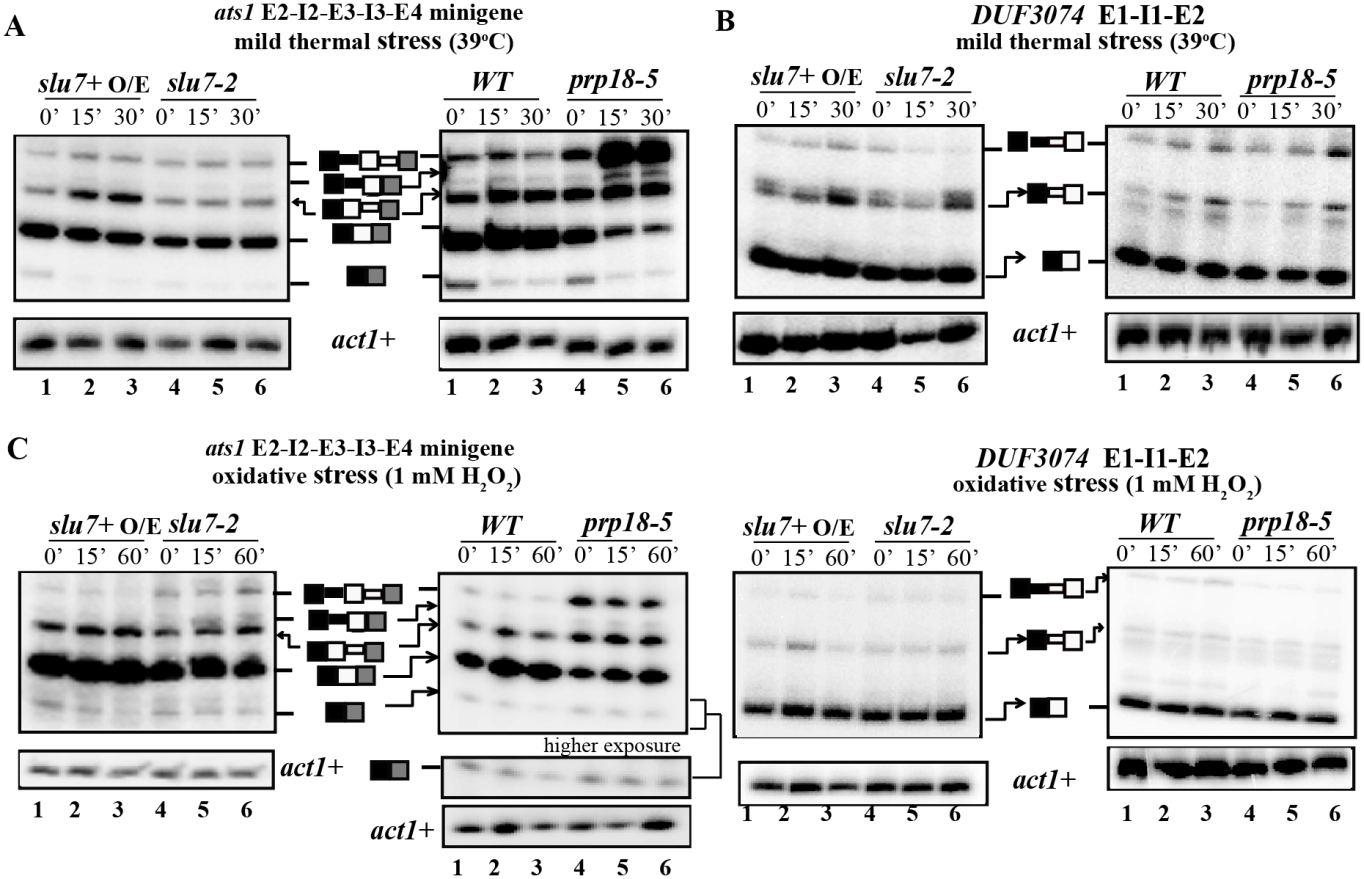
Effect of heatshock and oxidative stress on alternative splicing of *ats1*+ and DUF3074 introns. RT-PCR analyses for *ats1*+ E2-I2-E3-I3-E4 wild-type transcript and for cellular DUF3074 E1-I1-E2 transcript during mild thermal stress (39°C) (**A**), (**B**) and oxidative stress (1mM H_2_O_2)_ **(C)**, **(D)**. RNA was isolated from *slu7*+ O/E, *slu7-2*, *WT* and *prp18-5* cells, expressing wild-type or mutant versions of these splicing factors, subjected to stress treatment as detailed in materials and methods. Semi-quantitative RT PCR analyses were performed as in Fig 2. Intronless *act1+* transcript levels served as normalising control. The identity of the various splice forms are schematically indicated.

The physiological relevance of alternative splice site use and relationship to control of gene expression especially during environmental stress was examined. We studied splicing events in *ats1*+ and DUF3074 transcripts after subjecting fission yeast to mild thermal stress, oxidative and heavy metal stress. As described in [42], we used transcriptional upregulation of two CESR (core environmental stress response) genes *hsp16*+ and *hsp9*+ as hallmarks for the effectiveness of the subjected stress condition (S5 Fig). Since exposure to high temperature thermal stress (42°C for 15 and 30 min) resulted in an arrest of constitutive splicing of *tfIId+* intron 1 (S5A Fig), we tested the consequences of milder thermal stress of 39°C given for 15 and 30 min. This treatment did not cause constitutive splicing arrest as evidenced by the efficient splicing of *tfIId*+ I1 (S5B Fig). Hence, under these conditions the *in vivo* splicing status of *ats1*+ minitranscripts was studied in cells that express *slu7*+ O/E or cells that express the missense *slu7-2*. We found a rapid and nearly 2-fold increase in the *ats1*+ I3 retained mRNA isoform even in wild-type cells subjected to mild thermal stress (Fig 6A, left panel, lanes 1–3; right panel, lanes 1–3; S6A Fig). Within 15 min of mild thermal stress to wild-type cells a decrease in the levels of the low abundance *ats1*+ exon 3 skipped isoform occurred. Strikingly, the levels of *ats1*+ I3 retained mRNA did not increase after heat treatment of *slu7-2* cells that express the mutant protein (Fig 6A, left panel, lanes 4–6; S6A Fig). This data indicates that mild thermal stress-induced increase in *ats1*+ I3 retention is in part dependent on fully functional SpSlu7. As SpPrp18-5 mutant protein is unstable at temperatures higher than 37°C [37] even the mild 39°C thermal stress given to *prp18-5* cells arrested constitutive splicing of all introns in *ats1*+ (Fig 6A, right panel, lanes 4–6; S6B Fig). Such a strong splicing arrest was not seen in wild-type cells subjected to similar conditions. Taken together, our data indicate that in wild-type cells increase in *ats1*+ I3 retained form is a specific splicing response to mild thermal stress and this splicing profile change is dependent on SpSlu7. An alteration in splice isoform levels in response to mild thermal stress was also observed for DUF3074 intron 1. Here we observed increased utilisation of the alternative 3’ss splice site (Fig 6B, left panel, lanes 1–3; right panel, lanes 1–3; S6E Fig). The increased levels of alternative mRNA isoform for DUF3074 was also observed in *slu7-2* or *prp18-5* mutant cells suggesting the activities of these mutant proteins suffice to support choice of this non-canonical splice site during thermal stress (Fig 6B, left panel and right panel, lanes 4–6; S6E Fig). Alternatively it is possible that other splicing regulators could compensate or mask the reduced activity of mutant Slu7-2 or Prp18-5 proteins in the context of this intron. To examine if other environmental stresses also alter splicing patterns for *ats1*+ and DUF3074 transcripts we subjected wild-type cells, *slu7-2* and *prp18-5* mutant cells to oxidative and heavy metal stress. Wild-type cells subjected to oxidative stress showed reduced levels of *ats1*+ E3 skipped mRNA isoform with no change in I3 retained mRNA isoform unlike the effects of mild thermal stress (Fig 6C, left panel and right panel, lanes 1–3; S6C and S6D). However, the *ats1*+ E3 skipped mRNA levels were not reduced in *slu7-2* and *prp18-5* mutant cells subjected to oxidative stress (Fig 6C, left and right panel, lanes 4–6; S6C and S6D Fig). The data indicate that the oxidative stress induced decrease in E3 skipped form requires functional SpSlu7 and SpPrp18. The levels of the DUF3074 alternative mRNA made by use of a non-canonical 3’ss and the levels of the constitutive mRNA made by use of the canonical 3’ss were both unchanged in response to oxidative stress (Fig 6D, left and right panel, lanes 1–6; S6F Fig). The levels for various splice isoforms for the *ats1*+ E2-I2-E3-I3-E4 minitranscript are unchanged upon exposure to heavy metal stress (S7 Fig). These data reflect that splicing consequences of different stresses vary and indicate plausible roles for SpSlu7 and SpPrp18 in fine-tuning the levels of *ats1*+ splice isoforms specifically in response to mild thermal *versus* oxidative stress.

### Cotranscriptional elongation influences alternative splice site choice in *ats1*+

**Fig 7:**
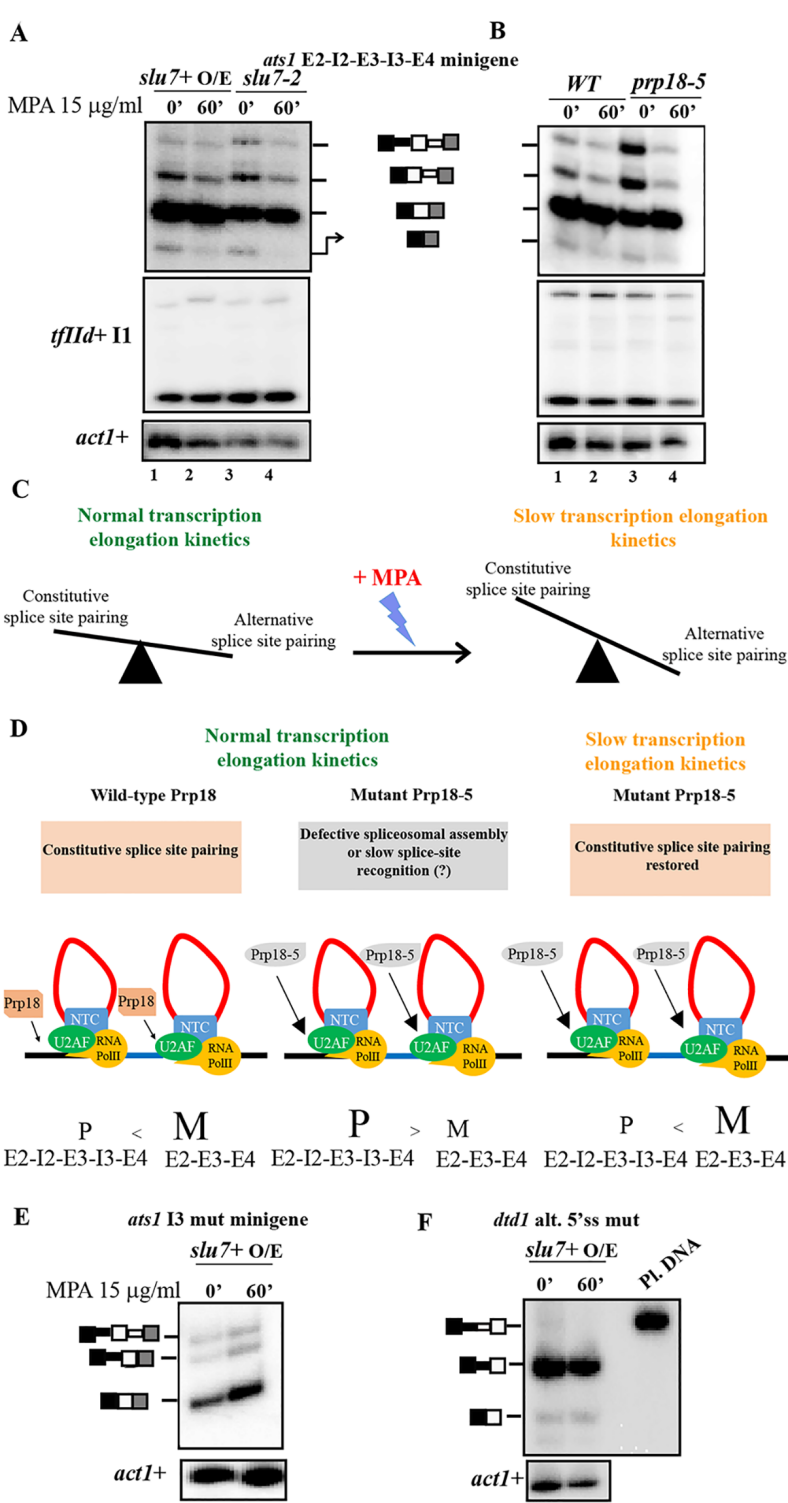
Effect of transcription elongation kinetics on alternative splice site choice. (**A, B**) Semi-quantitative RT PCR analyses of splicing status for wild-type *ats1*+ minitranscript in RNA from *slu7+* O/E, *slu7-2*, *WT* and *prp18-5* cells grown in the presence of 15 μg/ml mycophenolic acid for 60 min. The identity of the splice isoforms are schematically shown adjascent to the corresponding gel images. Splicing profile of constitutively spliced *tfIId+* I1 in the same RNA samples are shown in the middle panel. Intronless *act1*+ was used as normalising control. (**C**) Schematic diagram of the competition between constitutive and alternative splice site pairing under normal conditions and under reduced transcription elongation rates. (**D**) Proposed model showing the partial compensation of splicing defect in *prp18-5* strain for the *ats1+ E2-I2-E3-I3-E4* minitranscript upon inhibition of transcription elongation rate [addition of Mycophenolic acid (MPA)]. The exonic and intronic segments are indicated by black/blue and red solid lines respectively. Co-transcriptionally assembled spliceosomes are depicted with some key splicing factors like U2AF, NTC complex, omitting others for simplicity. The inefficient splicing in cells with Prp18-5 could be owing to slow recruitment (longer arrow) or reduced splicing catalysis under normal transcription rates which is compensated by slow transcription elongation rates (right panel). (**E**), (**F**) Splicing profile of *ats1* I3 mutant and *dtd1* alt. 5’ss mutant mintranscripts in *slu7*+ O/E cells subjected to MPA treatment.

Transcription elongation rates influence exon inclusion or skipping in both budding yeast [50] and fission yeast [28]. Therefore we questioned whether these mechanisms can contribute to the effects of SpSlu7 or SpPrp18 in the choice of weak splice sites and to exon skipping in *ats1*+. We tested possible co-ordination between transcription elongation kinetics and splicing factor activity by repressing transcription elongation rates by the supplementation of mycophenolic acid (MPA) to cultures. We found that one hour of MPA treatment abolished both the E3 skipped and I3 retained forms of *ats1+* in cells with wild-type Slu7 or Prp18 (Fig 7A and 7B, lane 2). This change in splice-site utilization was also seen in cells expressing mutant Slu7-2 or SpPrp18-5 proteins (Fig 7A and 7B, lane 4). Notably, the constitutive splicing of cellular *tfIId+* intron 1 was not affected by the conditions of MPA treatment given here. Therefore, this MPA treatment regime possibly does not cause widespread splice site utilization changes. Thus the *ats1+* E3 skipped form seen in wild-type cells is likely co-transcriptional and inhibition of transcription elongation rates further reduces the low levels of alternative splice site pairing that normally form the I3 retained and E3 skipped *ats1+* splice isoforms (Fig 7C).

As shown previously, the constitutive splicing of *ats1+* intron 2 and 3 is strongly dependent on SpPrp18 as manifested by the elevated level of pre-mRNA in untreated *prp18-5* cells (Fig 7B, lane 3 and Fig 3). Interestingly we find a reduction of the precursor levels in *prp18-5* cells given 1 hour of MPA treatment (Fig 7B, lane 4 and Fig 7D). This observation suggests that slower transcription elongation rates might partially compensate for the reduced splicing efficiency *ats1+* intron 3 in cells expressing mutant SpPrp18-5 protein. Further, we also explored if co-ordination between splice site strength and transcription elongation kinetics influences alternative splicing outcomes in *ats1+* and *dtd1*+. To this end we assessed the splicing of mutant *ats1+* and *dtd1+* minitranscripts (*ats1* I3 mut or *dtd1* alt. 5’ss mut minitranscripts) in wild-type *slu7*+OE cells. The splicing of these minitranscripts which harbour mutations that strengthen off-consensus splice sites followed the splicing profile observed in control untreated cells (Fig 7E and 7F). Thus these data support the view that splice site strength plays a primary role for the optimal choice of alternative non-consensus splice sites and subtle changes to transcription elongation kinetics contribute to fine tune transcript isoforms.

## Discussion

Fungal genomes, including *S*. *pombe*, are rich in transcripts with multiple short introns where splice site identification follows the intron-definition model [12]. In fission yeast apart from serine-arginine rich family of splicing factors [51, 52] that influence alternative splicing, little is known about the links between fission yeast splicing factors, environmental cues and regulated splicing. Here, we deduced low abundance yet reproducible alternative splicing events and then interrogated roles for the essential fission yeast splicing factors: SpSlu7 and SpPrp18. Both factors contribute to the low levels of intron 3 retained *ats1*+ isoform seen in wild-type cells; while SpPrp18 is required particularly for retention of *ats1*+ intron 2. Further both SpPrp18 and SpSlu7 could promote the utilisation of the non-canonical 5’ss of *dtd1*+ intron 1. The levels of the alternative isoforms of *ats1*+ and DUF3074 are regulated differently in response to mild thermal stress as compared to oxidative stress. Our data on the splicing profile of the multiple introns in fission yeast *ats1+* in wild-type cells and in the two mutants, *slu7-2* and *prp18-5* thus provide insights on roles for these spliceosome factors that in conjuction with transcription elongation rates generate transcript isoform diversity.

Intron retention is an alternatively spliced form that is widespread in lower eukaryotes, plants and more recent reports show such isoforms occur in the mammalian transcriptome [12, 53, 54]. We show that cellular RNA in wild-type fission yeast cells have distinctly different levels of intron retained *ats1+* mRNA isoforms (E2-E3-I3-E4 and E2-I2-E3-E4). The normal steady state levels of the *ats1+* I3 retained form requires functional SpSlu7 and SpPrp18. Intron retention in this case is related to its weak 5’ss (GUAGUG) as conversion to a better consensus sequence (GUAAGU) abrogated the I3 retained and also E3 skipped *ats1+* isoform even in wild-type cells. We further infer that the dependencies of non-canonical splice sites on functional splicing factors is intron-specific as *ats1*+ I2 retention is possibly more dependent on SpPrp18. Similar observations were deduced from ‘Intron Retention (IR) code’ in mammalian transcriptomes where retained introns had off-consensus *cis*-elements and were shorter [55, 54]. Together these data show that the splice site strength is closely related to intron retention in both fission yeast and human cells and our data show core spliceosomal factors have differential roles in intron retention.

The contribution of fission yeast splicing factors in promoting or repressing the use of alternative 5’ss or 3’ss in a given intron is not well understood. Such functions can contribute to the normal steady state levels of different spliced mRNA isoforms. The analysis of DUF3074 and *dtd1*+ transcript isoforms in cells where splicing factors SpSlu7 or SpSrp18 were depleted suggest roles for these proteins to promote the low level use of weak alternative splice sites in these transcripts. Our findings thus adds to the earlier reports on SR-protein kinase, Prp4 kinase and its effects on spliceosomal factors that regulate use of non-consensus splice sites [29, 30]. We observed that SpSlu7 is required for utilization of both canonical and non-canonical 5’ss in the *dtd1*+ intron 1. This function for fission yeast Slu7 in 5’ss choice contrasts with the role of its budding yeast and human counterparts where *in vitro* splicing assays with model mini-transcripts containing two tandem 3’ splice acceptor sites show Slu7 aids in the selection of a distal 3’ss [44, 34]. Interestingly, we found conversion of the sub-optimal downstream 5’ss of *dtd1*+ intron 1 into consensus sequence resulted in its use even after SpSlu7 depletion. Comparative intronic feature analysis to uncover underlying factors that may trigger predominant usage of the “strengthened alternative 5’ splice-site” over that of the constitutive splice site, revealed the variant intron arising from use of the mutant 5’ss is significantly smaller in length (67 versus 114 nts) with a higher percentage of AU residues particularly in the 5’ss-BrP region. Our previous work from global analyses of constitutive splicing indicated these intronic features (short introns, and high AU content) are correlated with decreased dependence for SpSlu7 [36] hence use of the “strengthened alternative 5’ splice-site” could be SpSlu7 independent. Similarly human PRPF8 factor independent splicing of introns normally retained in the CDC20 transcript was noted when the weak splice-sites that flank retained introns in CDC20 were strengthened [56]. Thus, sub-optimal splice sites can compete with the canonical splice sites and thereby contribute to the normal steady state cellular levels of spliced mRNA isoforms.

Interestingly, we find that the levels of alternatively spliced forms for *ats1*+, DUF3074 and *dtd1*+ transcripts are not at elevated levels in *upf1Δ*, a NMD pathway mutant suggesting that the cellular degradation of these spice isoforms is not mediated by this pathway alone. Alternate splicing though widespread in diverse fungal species such as budding yeast, fission yeast and *Cryptococcus neoformans* is predicted to largely create spliced RNA isoforms with limited coding potential [21, 19, 57]. As we do not detect alternate protein isoforms from *ats1*+ transcript isforms our data adds to other reports that indicate alternative splicing in *S*. *pombe*, and perhaps other fungi and lower eukaryotes, contributes very little to proteome diversity.

In budding yeast various environmental stresses including amino acid starvation, heat shock, hyper osmotic stress elicit diverse responses for intron containing genes [58–60]. In *Arabidopsis thaliana*, transcripts with stress-associated functions have a greater proportion of retained introns [61]. In fission yeast, heat shock increased exon skipping in the *alp41*+ transcript and cold shock had similar effects on the *qcr10*+ transcript [18]. We show wild-type cells given mild thermal stress or oxidative stress had stress-specific effects on the levels of *ats1*+ I3 retained and E3 skipped isoforms without change in the splicing efficiency for constitutively spliced introns. Mild thermal stress, rather than oxidative stress, caused accumulation of the *ats1*+ I3 retained isoform. Yet both stresses caused lowered levels of *ats1*+ E3 skipped form. These dynamic changes in splice isoforms were dependent on fully functional SpSlu7 as cells with the mutant Slu7-2 protein when given mild thermal stess do not exhibit these rapid changes in ratios of various splice isoforms. Further, we note that cells at logarithmic versus stationary growth phases have different ratios of *dtd1*+ mRNA isoforms with the choice of the sub-optimal 5’ss in intron 1 being distinctly increased during the stationary phase. These growth phase-specific and intron-specific effects are in line with findings from *Cryptococcus n*. *neoformans* wherein alternative splice sites in transcripts from CNH03510, exhibit varied profiles in exponential or stationary phase of growth [57]. The emerging studies implicate a complex interplay of *cis* and *trans* acting elements with different environmental cues that impinge on splicesome assembly and splice site utilization. For example, our finding of increased utilisation of alternate 3’ss in DUF3074 intron 1 in response to mild thermal stress may arise from an increased accessibility of the alternate 3’ss acceptor as was shown for *APE2* in heat stressed budding yeast cells [62]. In higher eukaryotes severe thermal stress inhibits splicing either by dephosphorylation of the mammalian SRSF10 causing splicing inhibiton or by sequesteration of selected splicing factors to nuclear stress bodies [63]. Nuclear concentration of different human splicing proteins are altered in diverse ways and this could variably modulate alternative splicing [64, 65].

In fission yeast overexpression of the SR protein Srp2 can suppress exon skipping defects of fission yeast mutants in U2AF23, U2AF59 and Bpb1 [28]. Also the association between fission yeast SR protein Srp2 and U2AF^23^ [66] and NTC component Cdc5 [67] are known. Interactions between early acting splicing factors in pre-catalytic spliceosomes (U2AF^59^ and Prp1), SpPrp18 and SpSlu7 are reported in our previous studies [36, 35]. Affinity capture studies of NTC complex factors Cdc5 and Cwf16, factors involved in spliceosomal activation and early recognition of branch point and 3’ss sequences respectively, also show SpSlu7 association with these factors [68, 28]. These interactions between Srp2, early assembling splicing factors and SpSlu7 are consistent with our findings that SpSlu7 influences alternative 5’ splice site choice.

Multiple studies have delineated the impact of chromatin organization upon alternative splicing [69, 70]. Additionally Brm, the catalytic component of the Swi/SNF remodeller interacts with U5 snRNP to influence splicing outcomes [69]. Our experiments on splicing profiles of *ats1+* introns in conditions where we perturbed transcription elongation by MPA addition show that co-transcriptional mechanisms promote generation of certain alternative splice-isoforms. Partial compensation of the poor splicing of *ats1+* introns in *prp18*-5 cells could be achieved when transcription elongation rates were reduced. These findings again support the very early association and interactions of SpPrp18 perhaps during spliceosome assembly on nascent elongating transcripts. While genetic or physical interactions of SpPrp18 with transcription machinery is not known, we have reported the genetic suppression interaction mutations in U2AF^59^, a very early acting splicing factor [37]. Yet interestingly we find that effects of mutations that strengthen off-consensus splice elements in *ats1+* and *dtd1+* introns are not reversed by compromising transcription elongation or when splicing factor activity was reduced. Therefore, we deduce that splice-site strength plays a greater role determining the use (or not) of a splice-site than does subtle changes to transcription elongation kinetics. Further, the activity of the core spliceosomal factors like SpSlu7 or SpPrp18 becomes crucial for utilization of weak splice site elements. Thus the interplay between transcription elongation rates of each transcript and *cis* elements in their introns dictates their dependency on splicing factors and thereby maintains the steady state levels of various transcript isoforms. Apart from directly regulating splicing catalysis, a splicing factor can also regulate intron retention events by indirect ways such as regulation of cellular localisation or transcript stability. For e.g., *ts* mutants in the essential budding yeast *MSL5* gene, encoding splicing factor ScSF1/BBP, although do not cause defective splicing, exhibit increase in cytoplasmic leakage of unspliced mRNAs suggesting a dual role for this factor in nuclear pre-mRNA retention and splicing [71]. Similarly, deletion of budding yeast *PRP18* gene increased the cytoplasmic leakage of unspliced pre-mRNAs whose nuclear retention was mediated by Mlp1, a perinuclear protein [72]. An example of the role of intron *cis* element in deciding the fate of the transcript isoform is the *C*. *neoformans CAS3* gene whose multiple introns regulate its expression mediated by the activity of poly-A binding protein Pab2 and exosome member Rrp44 [73]. The early splicing arrest on depletion of *slu7-2* and *prp18-5* missense mutants, their genetic interactions with early acting Prp1 and U2AF^59^, and as yet unknown possible interactions with other gene regulatory processes like transcription and chromatin organization are in line with the role of SpSlu7 and SpPrp18 in influencing alternative splicing fates (Fig 7C and 7D). These studies offer some testable mechanisms for future research that links environmental stresses to regulated splicing and the role of specific splicing factors such as SpSlu7 and SpPrp18 for splicing of short introns in fission yeast and other fungi.

## Supporting information

S1 Fig **(A)** Flowchart summarizing the strategy adopted to identify exon skipping events consistently observed in wild-type cells from splicing microarray and different RNA sequencing datasets. **(B)** Methodology adopted to choose candidates and experimentally verify the use of non-canonical/alternative splice sites in these transcripts.
(TIF)

S2 Fig Determination of identity corresponding to various cDNA products of *ats1+* E2-I2-E3-I3-E4 by sequence analysis. **(A)** Exon intron architecture of *ats1+* E2-I2-E3-I3-E4 minigene driven by heterologous *tbp1* promoter cloned in pDBlet shuttle vector (exon and intron sizes in nts are indicated in brackets). **(B)** Tracer labelled semi-quantitative RT-PCR analysis using exon 2 FP and vector-specific T7 RP in wild-type sample transformed with pDBlet *ptpb1 ats1+* E2-I2-E3-I3-E4 and the schematic representation of the identity the various cDNA amplicons from sequencing studies is indicated to the right. **(C)** Sequence chromatographs corresponding to *ats1+* exon 3 skipped mRNA (E2-E4), *ats1+* intron 2 retained mRNA (E2-I2-E3-E4) and *ats1+* intron 3 retained mRNA (E2-E3-I3-E4).
(TIF)

S3 Fig (**A**) Western blotting analysis of SpSlu7 levels in cell lysates of *slu7-2* strain grown in the presence and absence of thiamine using SpSlu7 polyclonal antisera (1:2500) as described in the Materials and methods. Coomassie-stained gel post-transfer serves as the loading control. (**B**) In vivo splicing analysis of *tfIId*+ intron 1 in the RNA samples of *WT* and *prp18-5* strains grown in the presence of absence of thiamine taken up for splicing assessment of *dtd1*+ I1 and DUF3074 I1 in Figs 4B and 5B.
(TIF)

S4 Fig Relative abundance of alternative splice isoforms in some RNA surveillance mutants. **(A)** Wild-type Fy527, NMD mutant *upf1Δ*, exosome mutant *dis3-54* strains were grown at 30°C. Constitutive and alternative splicing profile of *ats1*+ E2-I2-E3-I3-E4 minigene were assessed by semi-quantitative radiolabelled RT-PCR assays using T7 primer to selectively detect plasmid borne *ats1*+ minitranscripts. Intronless *act1*+ was used as normalising control. **(B)** Alternate use of canonical and non-canonical 3’ ss of DUF3074 intron 1. **(C)** Alternate use of non-canonical 5’ss of *dtd1*+ intron 1. After densitometric analysis of the various spliced products, the normalised levels of pre-mRNA, constitutive mRNA and alternate mRNA were plotted as bar graphs in both **(B)** and **(C)**. Intronless *act1*+ was used as normalising control. **(D)** Cloning of *ats1*+ wild-type gene in fission yeast expression plasmid pREP42 to express SpAts1 protein with N-terminal HA tag. Schematic of the peptides arising out of the various alternative splice events are represented along with the predicted molecular weight (calculated using EXPASY Translate) (left panel).Western blotting analysis of SpAts1 isoforms using anti-HA antibody (antiHA12CA5, Roche, 1:2500) (right panel).
(TIF)

S5 Fig Expression of marker genes to validate response to environmental stresses. (**A, B**) Semi-quantitative analysis of transcript levels of *hsp16*+, a heatshock responsive gene, at varying time points of both strong (42°C) and mild (39°C) thermal stress. Splicing status of intron 1 of constitutively expressed *tfIId*+ transcript. *act1*+ transcript served as the normalising control. (**C**) Gene expression analysis of *hsp9*+, an oxidative stress marker gene, at increasing time points of oxidative stress (1mM H_2_O_2_) along with the *in vivo* splicing assessment of *tfIId*+ intron 1.
(TIF)

S6 Fig Quantification of the various splice forms of *ats1*+ and DUF3074 in response to environmental stresses. **(A, B)** Data from the densitometric analysis of various isoforms of *ats1*+ in *slu7*+ O/E, *slu7-2*, *WT* and *prp18-5* cells subjected to mild thermal stress as shown in Fig 6A was plotted as bar graphs. The *p* value was calculated by unpaired student’s *t* test and asterisk denotes *p<0*.*05*. (**C, D**) Densitometric analysis of various isoforms of *ats1*+ in *slu7*+ O/E, *slu7-2*, *WT* and *prp18-5* cells subjected to oxidative stress for indicated time points as shown in Fig 6C. (**E, F**) Bar graphs plotting the levels of alternate mRNA of DUF3074 in *slu7*+ O/E, *slu7-2*, *WT* and *prp18-5* cells subjected to both thermal and oxidative stress for indicated time points as shown in Fig 6B and 6D.
(TIF)

S7 Fig Heavy metal stress-specific effects on alternative splicing. Effect of heavy metal stress on alternative and constitutive splicing of *ats1*+ E2-I2-E3-I3-E4 wild-type minitranscript. The analyses were performed in RNA from *slu7+* O/E, *slu7-2*, *WT* and *prp18-5* exposed to 0.5 mM CdS0_4_ (as detailed in Materials and methods). Intronless *act1*+ is used as normalising control.
(TIF)

S1 Table Oligonucleotides used for the study. (DOCX)

S2 Table List of 104 exon skipping events in wild-type fission yeast cells identified from the publically available NGS transcriptome data. The exon skipping events were identified using the alternate splice junction probes used in splicing-sensitive microarray platform. The sequence reads corresponding to these junctions in the two NGS transcriptome data [16, 34] as well as the raw intensities in the two replicates of *slu7*+O/E -T samples are indicated. The exon skipping events marked in cyan colour denote the ones for which the microarray raw intensity value is below 10 and not considered further.
(DOCX)

S3 Table List of twenty custom-designed probes to detect alternate mRNA isoforms arising from altered use of splice sites. In order to detect the ten isoforms formed due to the utilisation of altered 5’ss (donors) or 3’ss (acceptors) each, as reported in [16], twenty probe sequences were custom designed and incorporated in the SpPrp18 microarray platform [32].
(DOCX)
