# Supplementary figures and images for: Functions for fission yeast splicing factors SpSlu7 and SpPrp18 in alternative splice-site choice and stress-specific regulated splicing
Source: PLoS One. 2017 Dec 13;12(12):e0188159. doi: 10.1371/journal.pone.0188159 (PMC5728500; doi:10.1371/journal.pone.0188159)

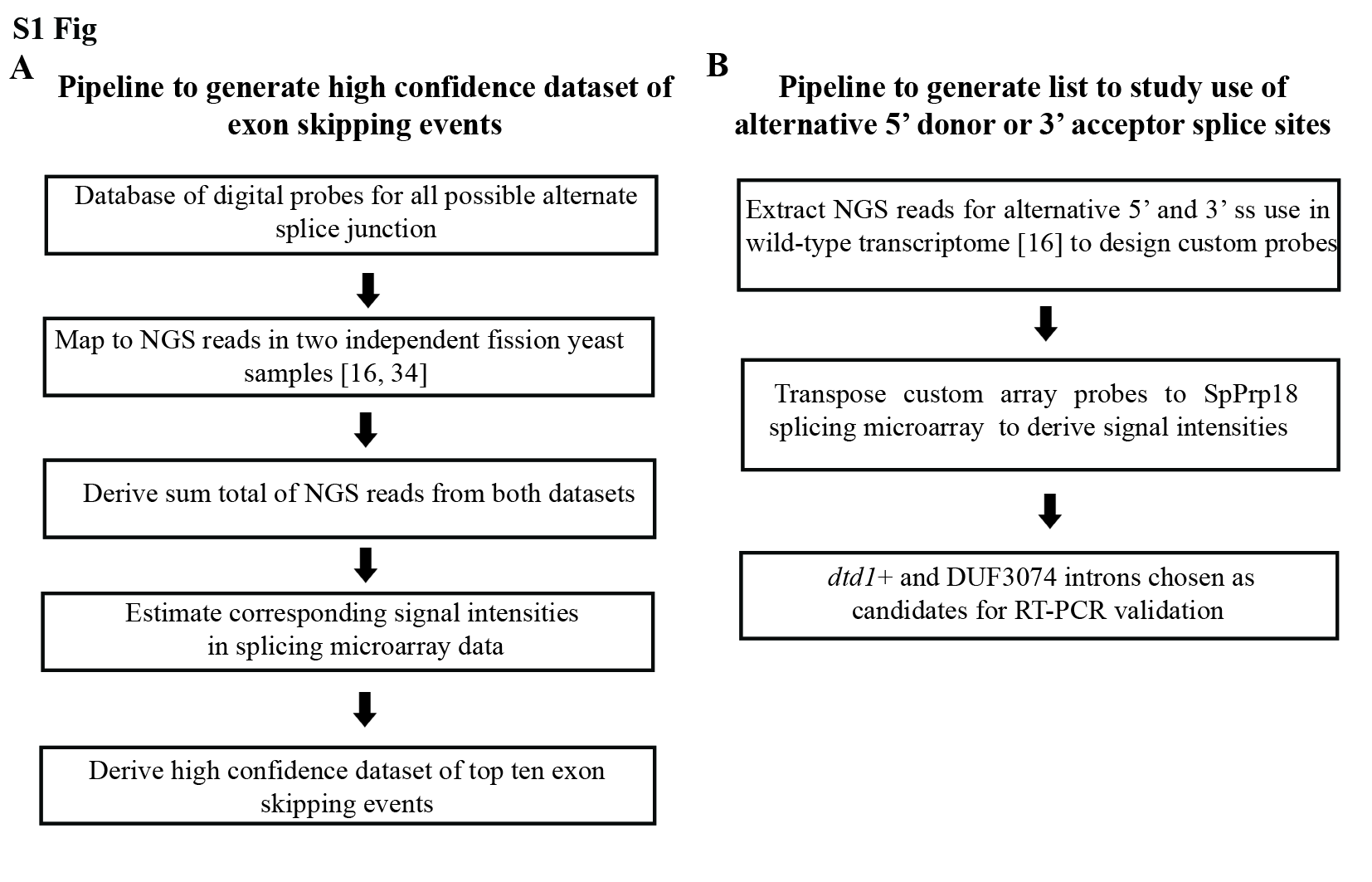

Supplement: S1 Fig — (A) Flowchart summarizing the strategy adopted to identify exon skipping events consistently observed in wild-type cells from splicing microarray and different RNA sequencing datasets. (B) Methodology adopted to choose candidates and experimentally verify the use of non-canonical/alternative splice sites in these transcripts. (TIF) [file pone.0188159.s001.tif]

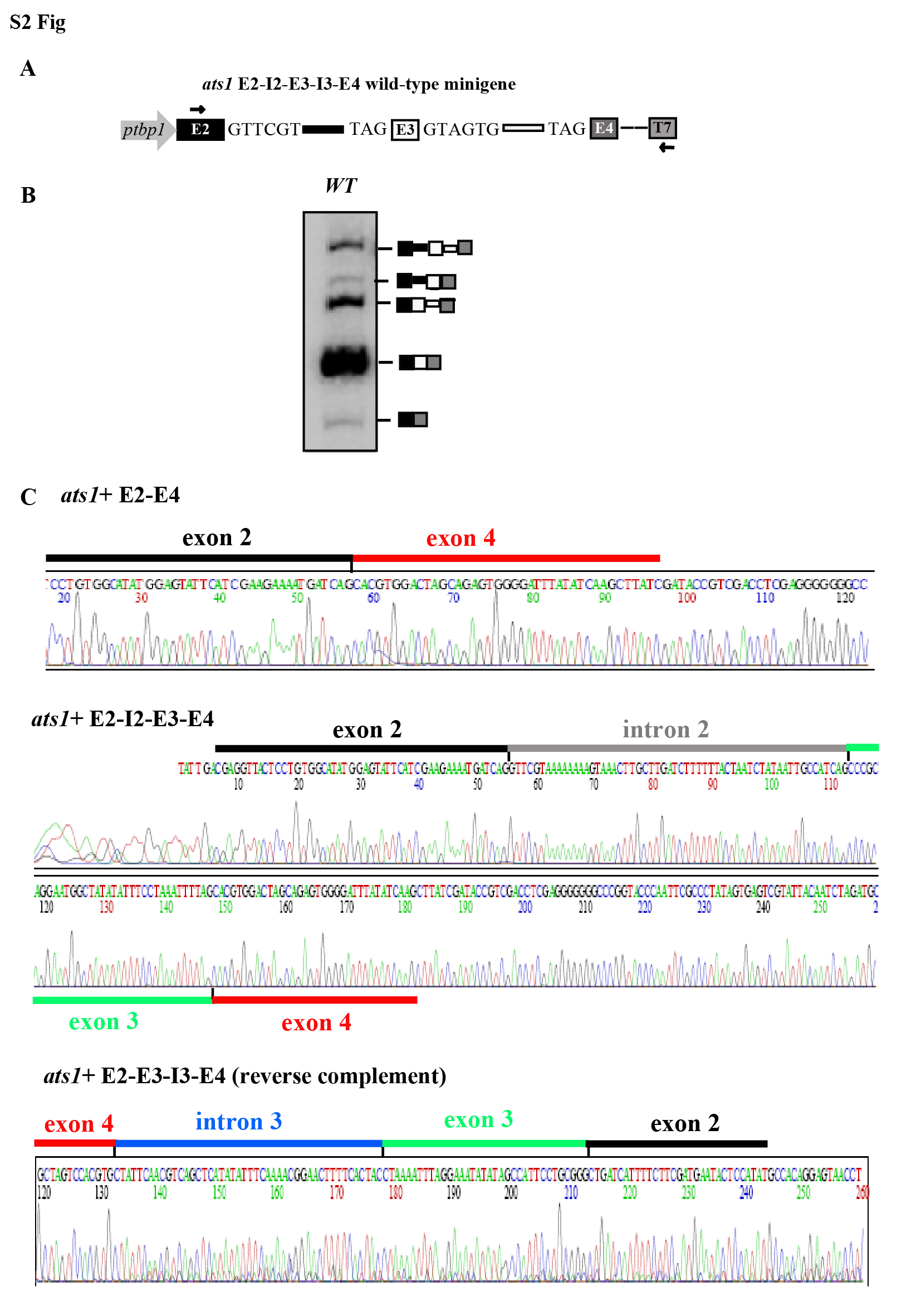

Supplement: S2 Fig — (A) Exon intron architecture of ats1+ E2-I2-E3-I3-E4 minigene driven by heterologous tbp1 promoter cloned in pDBlet shuttle vector (exon and intron sizes in nts are indicated in brackets). (B) Tracer labelled semi-quantitative RT-PCR analysis using exon 2 FP and vector-specific T7 RP in wild-type sample transformed with pDBlet ptpb1 ats1+ E2-I2-E3-I3-E4 and the schematic representation of the identity the various cDNA amplicons from sequencing studies is indicated to the right. (C) Sequence chromatographs corresponding to ats1+ exon 3 skipped mRNA (E2-E4), ats1+ intron 2 retained mRNA (E2-I2-E3-E4) and ats1+ intron 3 retained mRNA (E2-E3-I3-E4). (TIF) [file pone.0188159.s002.tif]

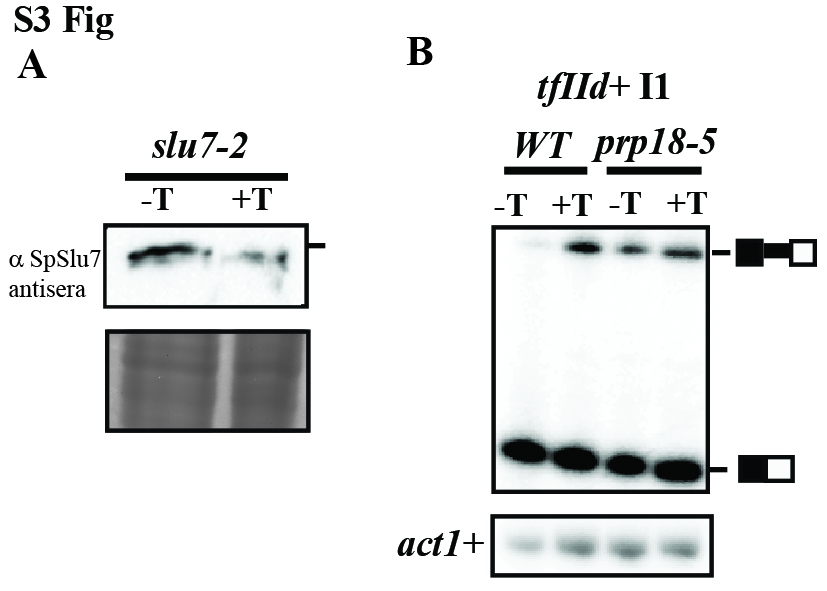

Supplement: S3 Fig — (A) Western blotting analysis of SpSlu7 levels in cell lysates of slu7-2 strain grown in the presence and absence of thiamine using SpSlu7 polyclonal antisera (1:2500) as described in the Materials and methods. Coomassie-stained gel post-transfer serves as the loading control. (B) In vivo splicing analysis of tfIId+ intron 1 in the RNA samples of WT and prp18-5 strains grown in the presence of absence of thiamine taken up for splicing assessment of dtd1+ I1 and DUF3074 I1 in Figs 4B and 5B. (TIF) [file pone.0188159.s003.tif]

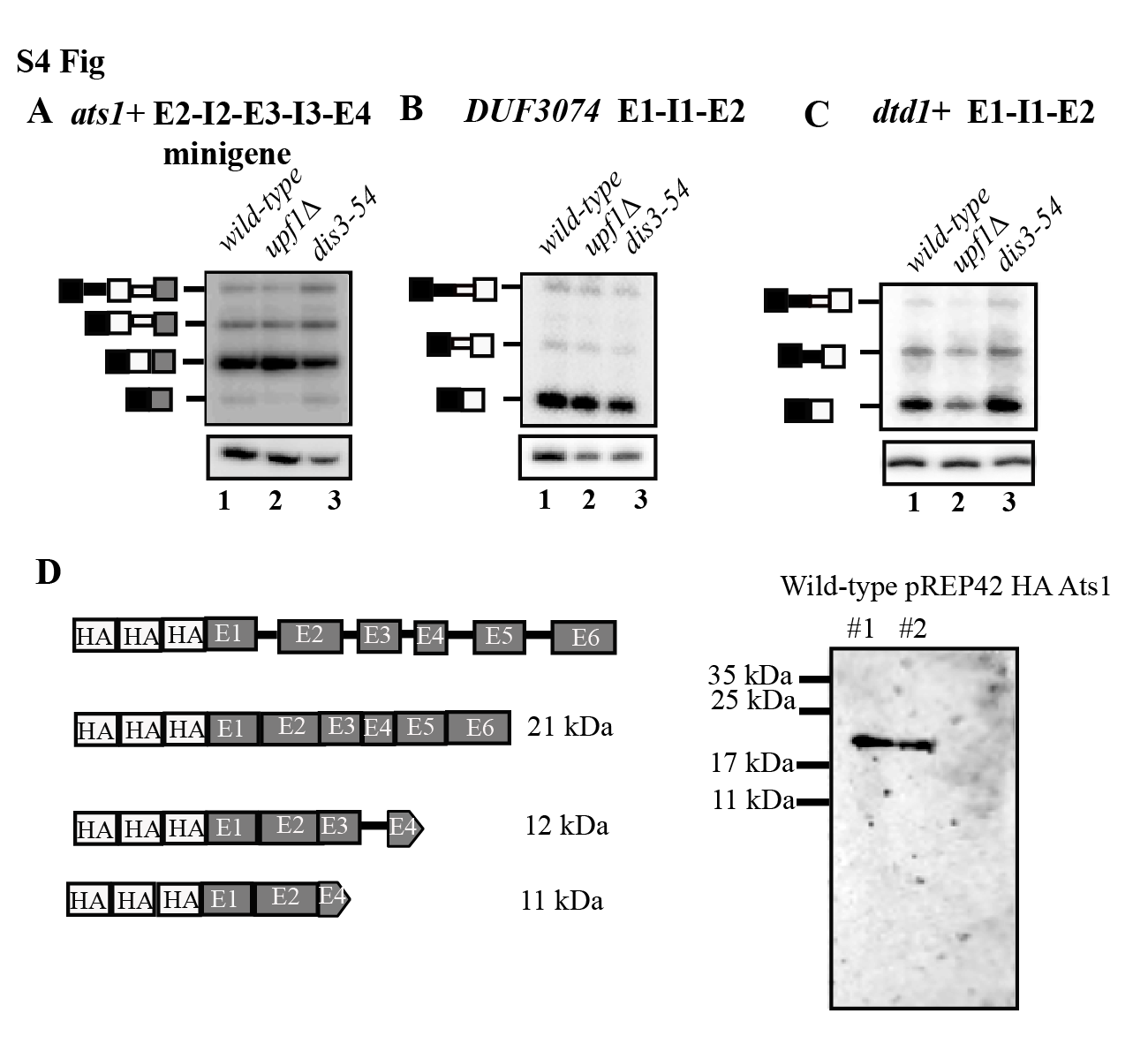

Supplement: S4 Fig — (A) Wild-type Fy527, NMD mutant upf1Δ, exosome mutant dis3-54 strains were grown at 30°C. Constitutive and alternative splicing profile of ats1+ E2-I2-E3-I3-E4 minigene were assessed by semi-quantitative radiolabelled RT-PCR assays using T7 primer to selectively detect plasmid borne ats1+ minitranscripts. Intronless act1+ was used as normalising control. (B) Alternate use of canonical and non-canonical 3’ ss of DUF3074 intron 1. (C) Alternate use of non-canonical 5’ss of dtd1+ intron 1. After densitometric analysis of the various spliced products, the normalised levels of pre-mRNA, constitutive mRNA and alternate mRNA were plotted as bar graphs in both (B) and (C). Intronless act1+ was used as normalising control. (D) Cloning of ats1+ wild-type gene in fission yeast expression plasmid pREP42 to express SpAts1 protein with N-terminal HA tag. Schematic of the peptides arising out of the various alternative splice events are represented along with the predicted molecular weight (calculated using EXPASY Translate) (left panel).Western blotting analysis of SpAts1 isoforms using anti-HA antibody (antiHA12CA5, Roche, 1:2500) (right panel). (TIF) [file pone.0188159.s004.tif]

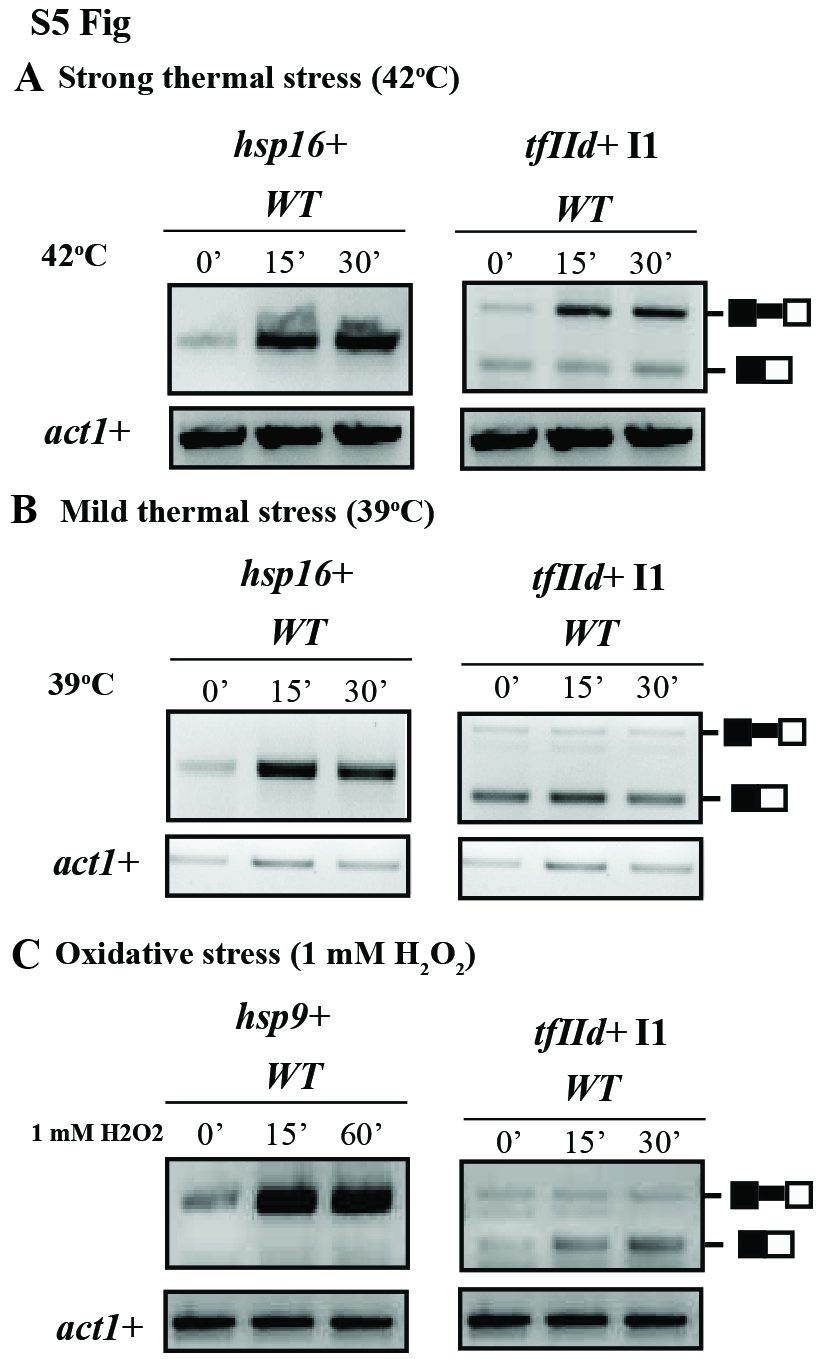

Supplement: S5 Fig — (A, B) Semi-quantitative analysis of transcript levels of hsp16+, a heatshock responsive gene, at varying time points of both strong (42°C) and mild (39°C) thermal stress. Splicing status of intron 1 of constitutively expressed tfIId+ transcript. act1+ transcript served as the normalising control. (C) Gene expression analysis of hsp9+, an oxidative stress marker gene, at increasing time points of oxidative stress (1mM H2O2) along with the in vivo splicing assessment of tfIId+ intron 1. (TIF) [file pone.0188159.s005.tif]

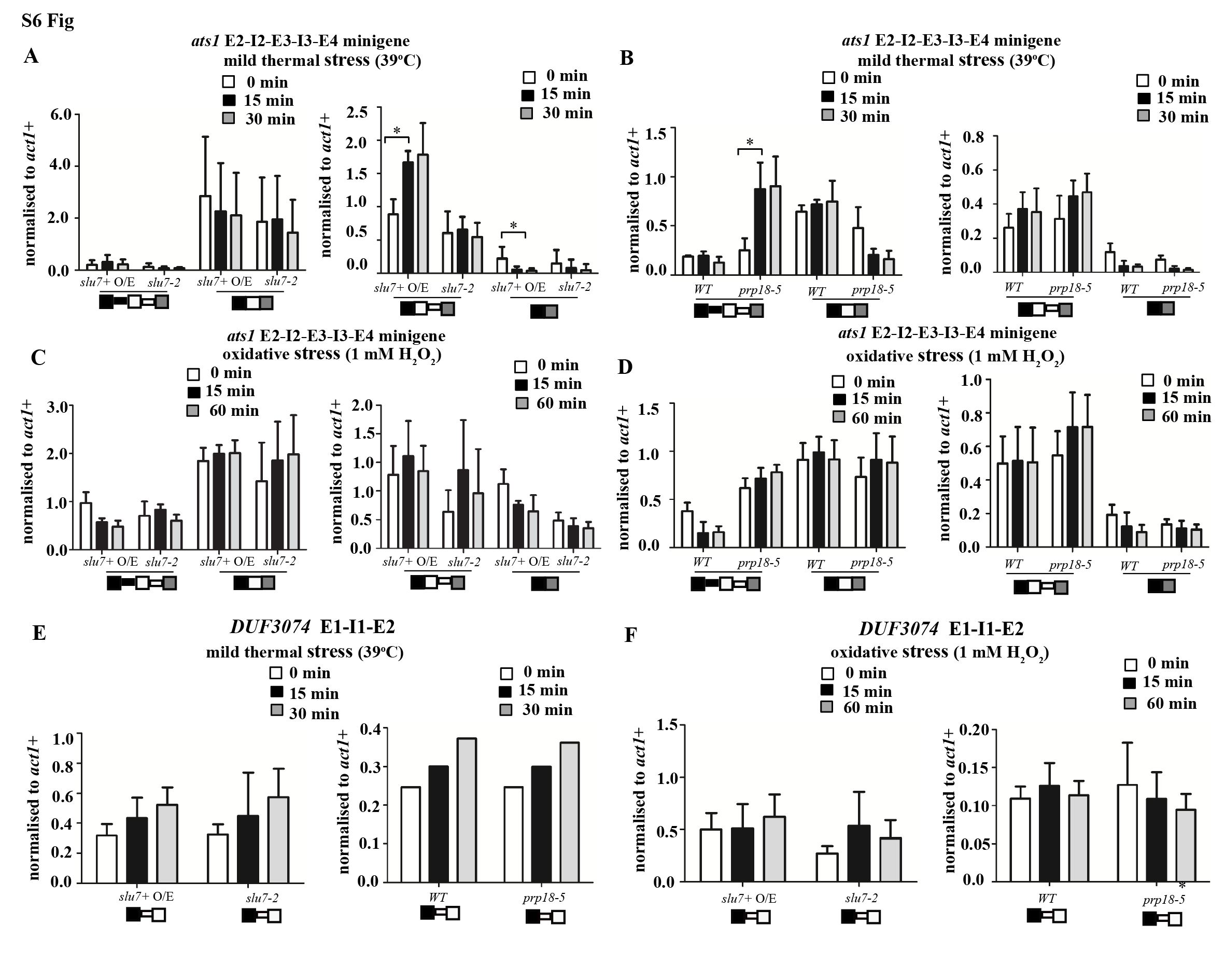

Supplement: S6 Fig — (A, B) Data from the densitometric analysis of various isoforms of ats1+ in slu7+ O/E, slu7-2, WT and prp18-5 cells subjected to mild thermal stress as shown in Fig 6A was plotted as bar graphs. The p value was calculated by unpaired student’s t test and asterisk denotes p<0.05. (C, D) Densitometric analysis of various isoforms of ats1+ in slu7+ O/E, slu7-2, WT and prp18-5 cells subjected to oxidative stress for indicated time points as shown in Fig 6C. (E, F) Bar graphs plotting the levels of alternate mRNA of DUF3074 in slu7+ O/E, slu7-2, WT and prp18-5 cells subjected to both thermal and oxidative stress for indicated time points as shown in Fig 6B and 6D. (TIF) [file pone.0188159.s006.tif]

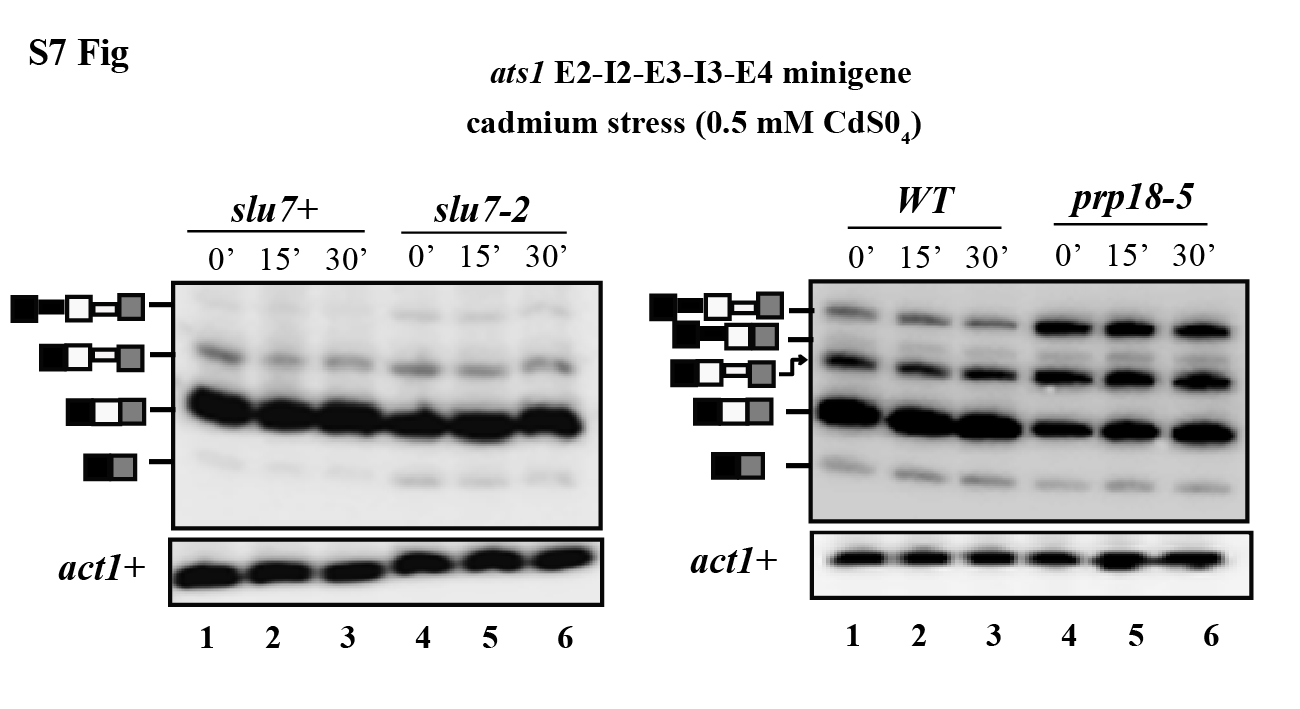

Supplement: S7 Fig — Effect of heavy metal stress on alternative and constitutive splicing of ats1+ E2-I2-E3-I3-E4 wild-type minitranscript. The analyses were performed in RNA from slu7+ O/E, slu7-2, WT and prp18-5 exposed to 0.5 mM CdS04 (as detailed in Materials and methods). Intronless act1+ is used as normalising control. (TIF) [file pone.0188159.s007.tif]
