# Supplementary material for: Functions for fission yeast splicing factors SpSlu7 and SpPrp18 in alternative splice-site choice and stress-specific regulated splicing
Source: PLoS One. 2017 Dec 13;12(12):e0188159. doi: 10.1371/journal.pone.0188159 (PMC5728500; doi:10.1371/journal.pone.0188159)
Supplement: S1 Table — (DOCX) [file pone.0188159.s008.docx]

**S1 Table. Oligonucleotides used for the study.**

| **Primer Name** | **Sequence** |
| --- | --- |
| spact1 FP | 5’ GCTGCTCAATCTTCCTCCCTTG 3’ |
| spact1 RP | 5’ GGTCCGCTCTCATCATACTCTT 3’ |
| ats1 E2 FP | 5’ AAGATCAAGTGGAAGCGACA 3’ |
| ats1 E4 RP | 5’ ATAAATCCCCACTCTGCTAGT 3’ |
| Ats1 I3 mut FP | 5’ CCTAATTTTAGGTAAGTAAAAGTTCCG 3’ |
| Ats1 I3 mut RP | 5’ CGGAACTTTTACTTACCTAAAATTAGG 3’ |
| DUF3074 E1FP | 5' TACATGGTATGGTAGGGTGT 3’ |
| DUF3074 E2RP | 5’ AGTTTCCAATTGATGTGCTG 3’ |
| dtd1+ E1FP | 5’ ATGAAGGCAGTAATTCAGC 3’ |
| dtd1+ E2RP | 5’CTTGTTGAATGGCAGAAAC 3’ |
| Dtd1 mut FP | 5’ GGACGGATTTTATAATCGGTAAGTAGAAAAT 3’ |
| Dtd1 mut RP | 5’ ATTTTCTACTTACCGATTATAAAATCCGTCC 3’ |
| T7 RP | 5’ GTAATACGACTCACTATAGGGC 3’ |

Underlined sequences indicate the mutated 5’ss of *ats1*+ and *dtd*1+.
