# Supplementary material for: Functions for fission yeast splicing factors SpSlu7 and SpPrp18 in alternative splice-site choice and stress-specific regulated splicing
Source: PLoS One. 2017 Dec 13;12(12):e0188159. doi: 10.1371/journal.pone.0188159 (PMC5728500; doi:10.1371/journal.pone.0188159)
Supplement: S2 Table — The exon skipping events were identified using the alternate splice junction probes used in splicing-sensitive microarray platform. The sequence reads corresponding to these junctions in the two NGS transcriptome data [16, 34] as well as the raw intensities in the two replicates of slu7+O/E -T samples are indicated. The exon skipping events marked in cyan colour denote the ones for which the microarray raw intensity value is below 10 and not considered further. (DOCX) [file pone.0188159.s009.docx]

**S2 Table**. **List of 104 exon skipping events in wild-type fission yeast cells identified from the publically available NGS transcriptome data.** The exon skipping events were identified using the alternate splice junction probes used in splicing-sensitive microarray platform. The sequence reads corresponding to these junctions in the two NGS transcriptome data [16, 34] as well as the raw intensities in the two replicates of *slu7+* -T samples are indicated. The exon skipping events marked in cyan colour denote the ones for which the microarray raw intensity value is below 10 and not considered further.

| \|  \|  \|  \|  \| **WT Microarray dataset** \| \| **RNA sequencing datasets** \| \| \| \| \| \| --- \| --- \| --- \| --- \| --- \| --- \| --- \| --- \| --- \| --- \| --- \| \| **GeneName** \| **5' exon** \| **3' exon** \| **splice junction probe sequence (5'-3')** \| **slu7+ -T#1** \| **slu7+ -T #1** \| **MM1** \| **MM2** \| **SRX040570** \| **SRX040571** \| **Total** \| \| SPAC6F6.07c \| 1 \| 3 \| ATTCCTCAAGTTCGTTTCATCACTGGTCTTGCTCCCGAGCTCCCCGAGGA \| 80031.93 \| 50799.895 \| 0 \| 0 \| 18 \| 18 \| 36 \| \| SPAC6F6.07c \| 1 \| 4 \| ATTCCTCAAGTTCGTTTCATCACTGGCTGTTTCCGTCCGCAAGCATTTGG \| 61870.848 \| 40849.246 \| 0 \| 0 \| 0 \| 3 \| 3 \| \| SPBC19G7.03c \| 1 \| 3 \| CGGATCTCTCGCTCGTGCAGGAAAGATGAATCCTTCTTCTTAAGCAGTTC \| 18797.605 \| 11396.047 \| 0 \| 0 \| 17 \| 24 \| 41 \| \| SPBC119.02 \| 1 \| 3 \| TTGGCAAGCTACAATCATGGGTCCTGTAAACTTTACAACCAGAATCTATC \| 12149.073 \| 14549.23 \| 0 \| 0 \| 2 \| 1 \| 3 \| \| SPAC16.02c \| 8 \| 10 \| CTCACCGTGAAAACCCTGGTGCTGGGATGCCGCTAATGAGCCCCTTCCCG \| 8407.14 \| 7558.701 \| 0 \| 0 \| 3 \| 0 \| 3 \| \| SPBC16C6.08c \| 1 \| 4 \| GCCAACCTAAGTCTCAAGACTCAAGAATGCATGGATGCACCAGATTGCAA \| 4264.973 \| 4909.746 \| 0 \| 0 \| 2 \| 5 \| 7 \| \| SPAC16.02c \| 7 \| 10 \| TCTCTCAGAAGATGTCAGTTGGCAGGATGCCGCTAATGAGCCCCTTCCCG \| 2019.777 \| 1714.117 \| 0 \| 0 \| 4 \| 3 \| 7 \| \| SPAC1002.07c \| 2 \| 4 \| AGTATTCATCGAAGAAAATGATCAGCACGTGGACTAGCAGAGTGGGGATT \| 1784.937 \| 1334.991 \| 0 \| 0 \| 6 \| 4 \| 10 \| \| SPBC16C6.08c \| 1 \| 3 \| GCCAACCTAAGTCTCAAGACTCAAGAAACCATTCAACCCCCTGAAGAAGT \| 1474.788 \| 1883.878 \| 0 \| 0 \| 6 \| 1 \| 7 \| \| SPAC16.02c \| 3 \| 5 \| CGCGATGCTCGTGATATCGTAAATGAGGGCAAAGAGTTTATGGGCAGCCG \| 1278.593 \| 952.948 \| 0 \| 0 \| 2 \| 1 \| 3 \| \| SPAC17G8.14c \| 2 \| 4 \| TACCCCCGAATTCATGGCTCCAGAACTTTTAACAAGAGATCCAAATCAAC \| 1064.988 \| 1213.26 \| 0 \| 1 \| 3 \| 0 \| 4 \| \| SPAC16.02c \| 5 \| 10 \| AGGGCAAAGAGTTTATGGGCAGCCGGATGCCGCTAATGAGCCCCTTCCCG \| 760.889 \| 630.376 \| 0 \| 0 \| 0 \| 4 \| 4 \| \| SPAC3F10.09 \| 1 \| 3 \| AAAACGGCTTTGCACACATGGCCTGGTAATAGTAACTTCTTGGCTGTTCC \| 606.327 \| 642.495 \| 0 \| 0 \| 5 \| 3 \| 8 \| \| SPCC1919.06c \| 1 \| 4 \| GAGGGCGCGTTACCACCATATTCAGTGGAACCGGGATCTTGCTCTACAGA \| 555.392 \| 1001.798 \| 0 \| 0 \| 0 \| 3 \| 3 \| \| SPBC19G7.17 \| 3 \| 5 \| AAACTGATACTCTGGACCCTATCAATGGACTTATGATTACCGGCTTGGCC \| 549.767 \| 413.04 \| 0 \| 0 \| 2 \| 2 \| 4 \| \| SPCC1672.12c \| 3 \| 6 \| TATGCTGCCTCTCTTGACAACAAAGGGAAAGAACCCGCTAGCGCTGAGAA \| 432.916 \| 469.894 \| 0 \| 0 \| 0 \| 6 \| 6 \| \| SPAC1687.21 \| 3 \| 5 \| AGTGTATACTGATTTAATTCGCGAGGCATGAACGTTGTAGAAGCCAAAAA \| 333.988 \| 278.049 \| 1 \| 0 \| 2 \| 0 \| 3 \| \| SPAC6F6.15 \| 5 \| 7 \| TATAAATCTCTAGCTCCTATGTACTGCATCTCTAGAAAAGGCCAAATCTT \| 331.554 \| 546.704 \| 0 \| 0 \| 1 \| 3 \| 4 \| \| SPAC732.01 \| 2 \| 6 \| TATTCTTCTTTTTTTGGTTTTGCAGCGGATTTATTCATCTCTCTGCTGGA \| 309.432 \| 343.998 \| 0 \| 0 \| 2 \| 1 \| 3 \| \| SPCC1235.15 \| 1 \| 3 \| ATGGGCTTTCCTCCCATTTTGGCCTGGGCCGATTTTAGTAAATTATTTCC \| 307.36 \| 492.494 \| 0 \| 0 \| 2 \| 1 \| 3 \| \| SPCC1739.09c \| 1 \| 4 \| GAGGAGCATGCAAAGCGTAGCAGTGATTGCAAGCATCAAGAGCATGCCAA \| 293.918 \| 386.228 \| 0 \| 0 \| 0 \| 4 \| 4 \| \| SPAC17G8.14c \| 3 \| 5 \| GGATGCGGTTTCGTTATTGAGAGGGGTGCATGTCCAACTTTGACACCTGT \| 293.581 \| 381.181 \| 0 \| 0 \| 2 \| 1 \| 3 \| \| SPCC1672.12c \| 2 \| 4 \| AAATGCTTCGCACTCTAGTAAATAGCCCGAGCTTTGACTCTTTTGGGACT \| 283.857 \| 286.972 \| 0 \| 0 \| 2 \| 1 \| 3 \| \| SPBC839.17c \| 2 \| 4 \| AAGACTTTCCCAAACCTGGTGACAGGTACTTTAACCAATGGTAAAAAGTT \| 221.991 \| 261.381 \| 0 \| 0 \| 0 \| 4 \| 4 \| \| SPBC2D10.20 \| 1 \| 6 \| CGTAGAATTGCTAAAGAACTTGCTGATTATCCATTTCGCCCCCCTAAAAT \| 221.423 \| 289.448 \| 0 \| 0 \| 2 \| 1 \| 3 \| \| SPAC1782.07 \| 1 \| 3 \| GTGCTGCTGGAGGAAAAACTTATTTAGGTGGACCTAAACAAAAAGGAATC \| 219.488 \| 274.489 \| 1 \| 1 \| 0 \| 7 \| 9 \| \| SPBC1709.04c \| 2 \| 4 \| GGTTGTTTTCCAGTATAGTCCCGAAACAATTTTGTACAGGAGAAACGCTT \| 219.465 \| 154.913 \| 0 \| 0 \| 0 \| 5 \| 5 \| \| SPBC660.16 \| 1 \| 3 \| GAAGAATCATGTCACAAAAAGAAGTATTTTGGTCTCATCGGTTTGGCCGT \| 186.773 \| 470.81 \| 0 \| 0 \| 0 \| 3 \| 3 \| \| SPCC1442.08c \| 1 \| 4 \| CCTAAGCAGTTTACTTTTGGTACAGATTGCTTCCAAAGTTACATTGATTA \| 183.687 \| 284.032 \| 0 \| 0 \| 1 \| 4 \| 5 \| \| SPBC3E7.07c \| 1 \| 3 \| GGAAAATGCGGAAAATGCCGCCGAGTTTGCAGTAAAAGCTGTTGAACAGG \| 165.699 \| 134.492 \| 0 \| 3 \| 5 \| 9 \| 17 \| \| SPAC22F3.05c \| 2 \| 4 \| TTTGAAGTGCCTGTTGAACGAGGATGGGACATTGGGGGGCAGAAAACGCT \| 148.284 \| 80.977 \| 1 \| 4 \| 1 \| 5 \| 11 \| \| SPCC1442.08c \| 1 \| 5 \| CCTAAGCAGTTTACTTTTGGTACAGAATGGGTAGAACGCTGGGACGAACA \| 146.048 \| 187.013 \| 0 \| 0 \| 2 \| 2 \| 4 \| \| SPBC1709.14 \| 3 \| 5 \| GATGAACCTTTGATATACGAACAAGTCGATAGTGTTCGCGATGTATCGCA \| 138.427 \| 159.991 \| 1 \| 0 \| 2 \| 1 \| 6 \| \| SPAC3G6.03c \| 1 \| 3 \| CAGAAAGCAGTTATTTGAACAGATGGCCGCCGAAGAAGATTCACCAGACA \| 129.996 \| 199.99 \| 0 \| 0 \| 3 \| 3 \| 6 \| \| SPAPB1A10.10c \| 1 \| 3 \| TGGAAAAACATGTTTAATGAATCAGCTTTGGGATACTGCTGGTCAAGAAC \| 125.996 \| 128.996 \| 0 \| 0 \| 0 \| 4 \| 4 \| \| SPBC1271.11 \| 2 \| 4 \| GGTGGTCTTTCCGGATTAGTTGCAGGTTTTTCCTCTTTCTACAATAATTA \| 123.742 \| 92.749 \| 0 \| 0 \| 2 \| 1 \| 3 \| \| SPAC1687.21 \| 2 \| 5 \| TAGCTCTATGAAGCGATGCCGGGAGGCATGAACGTTGTAGAAGCCAAAAA \| 115.412 \| 107.184 \| 0 \| 0 \| 8 \| 61 \| 69 \| \| SPAC6F6.15 \| 2 \| 4 \| CGATTCGTTAAAGATCAATTTGATGCTGCTTTTTTGACTCAAACCCTTCC \| 98.537 \| 194.961 \| 0 \| 0 \| 12 \| 40 \| 52 \| \| SPBC2G2.03c \| 1 \| 3 \| ATCAGCCAGAATGTCATCCACAAAAGAGACGTGCTGCTGTTGAAAAGAAC \| 94.169 \| 114.93 \| 0 \| 0 \| 7 \| 6 \| 13 \| \| SPBC16E9.16c \| 1 \| 3 \| ACAGTAGCTATGCCTCTAGCAAAAACAAGATCGCTTCAACGCCGCCAAGT \| 81.075 \| 313.731 \| 0 \| 0 \| 1 \| 5 \| 6 \| \| SPAC1687.17c \| 1 \| 3 \| TAAGCCAAACTCCTCCGGTTACGAGTACTGGAGGGCAATAACTACATTCC \| 69.407 \| 76.03 \| 0 \| 0 \| 2 \| 2 \| 4 \| \| SPAC222.18 \| 1 \| 3 \| GGACACTAGGCCAGGCCTTTGAAAAGTATGCTTTCGTGGAATTTGAAGAG \| 62.915 \| 55.934 \| 0 \| 0 \| 2 \| 1 \| 3 \| \| SPBC16C6.08c \| 2 \| 4 \| GCCGATGAAAAAGAGGAGAAAAAGGAATGCATGGATGCACCAGATTGCAA \| 56.707 \| 82.384 \| 0 \| 1 \| 4 \| 3 \| 8 \| \| SPAC8E11.04c \| 2 \| 4 \| ATATTAAGTGGATCTTTCCCAATGCGTATGATATCTACAGTTTTGCGGAT \| 56.241 \| 72.497 \| 0 \| 0 \| 3 \| 1 \| 4 \| \| SPBC16H5.11c \| 1 \| 3 \| GATGAAGTAGCTTATCACCCTGAGGAAACCCATGTACGTCCCAGAAAACA \| 49.061 \| 66.951 \| 0 \| 2 \| 0 \| 1 \| 3 \| \| SPCC1739.15 \| 1 \| 6 \| GAGGGCGCATTACCTCCATATTCAGGAGGGATAGGAAATGCGATGATGGG \| 46.533 \| 60.541 \| 0 \| 0 \| 0 \| 4 \| 4 \| \| SPBC336.14c \| 2 \| 4 \| TGGATGTGCTTTTCGACATGATGGTTAAGAAAAAACTGAATGCTGCGTCG \| 45.113 \| 34.942 \| 0 \| 0 \| 3 \| 3 \| 6 \| \| SPAC630.11 \| 1 \| 3 \| AATTAAATATGTCCGACCTCAGGAAGTCCTGGCAGTAGGATTTATGCTGG \| 44.833 \| 51.137 \| 0 \| 0 \| 5 \| 2 \| 7 \| \| SPBC651.06 \| 4 \| 6 \| TTTCTCAATGAAAGATTAGAACACTTTACAAGAAGCTACCGCCTTTCCCT \| 39.721 \| 22.673 \| 0 \| 0 \| 4 \| 8 \| 12 \| \| SPAC1556.05c \| 1 \| 3 \| GGAACTAAGGGTGTATGTGTTTCAGGAAAACTGAGAAAAAGGCGTACAAC \| 39.2 \| 28.476 \| 0 \| 1 \| 0 \| 3 \| 4 \| \| SPAC1002.07c \| 1 \| 3 \| TATCAGTTTATTAAAGAATTGGCAGCCCGCAGGAATGGCTATATATTTCC \| 37.327 \| 23.779 \| 0 \| 0 \| 2 \| 1 \| 3 \| \| SPAC1687.21 \| 1 \| 3 \| CGACCAGAACAAACGCGGTATCTTAACAATCGCCCCTTATCTTGAGCTTA \| 29.091 \| 39.916 \| 0 \| 0 \| 0 \| 6 \| 6 \| \| SPBP4H10.08 \| 2 \| 5 \| TCACCCCTGAAAGAACTATGAAAACCCTATCGCATTCATTGGCGACCACT \| 27.695 \| 50.643 \| 0 \| 0 \| 0 \| 7 \| 7 \| \| SPBC651.06 \| 1 \| 6 \| CCTTCTTTACTCAAAAGTCGATCAATTACAAGAAGCTACCGCCTTTCCCT \| 23.637 \| 21.667 \| 0 \| 0 \| 0 \| 4 \| 4 \| \| SPBC9B6.10 \| 2 \| 4 \| ATAAGCTAGAACTTAGTGATGACAGTTCATCCAAATGTCGATAAAAAGTC \| 21.535 \| 25.692 \| 0 \| 0 \| 6 \| 4 \| 10 \| \| SPAC5D6.05 \| 1 \| 3 \| TTTTGGGTAGTTTACCGTCCTAAAGGTCAATGTACTTAGAAGGGAACTCA \| 21.407 \| 20.769 \| 0 \| 0 \| 2 \| 1 \| 3 \| \| SPAC1751.02c \| 1 \| 3 \| GCCAGAGCTCGGTCAGTCTGGAAAGTGCTCAATTTATGGTTCATAATGGT \| 19.799 \| 78.849 \| 0 \| 0 \| 0 \| 3 \| 3 \| \| SPAC2F3.17c \| 1 \| 3 \| TTAAGTTCTGGTGTCGACTATAAAGTCTTATATGTTTCTGCTTTAGATGA \| 19.696 \| 16.785 \| 0 \| 0 \| 0 \| 3 \| 3 \| \| SPAC4D7.09 \| 2 \| 5 \| TCGTGCTGTATCCCATCTTATAAAGATCGCCAAACTTACTCCAGAACAAC \| 19.352 \| 17.887 \| 0 \| 0 \| 0 \| 5 \| 5 \| \| SPCC1739.09c \| 3 \| 5 \| GCTAGTGCCAATGCGTATTATATTTCTTTGAAAATTTGCGCTTCAAAAAG \| 18.966 \| 14.367 \| 0 \| 0 \| 1 \| 2 \| 3 \| \| SPAC27D7.08c \| 5 \| 7 \| GGTTGATAAATTGCGTGAACAAAATGAACTTATACGTCCTTCTTCAACTT \| 18.225 \| 21.196 \| 0 \| 0 \| 4 \| 2 \| 6 \| \| SPBC19G7.10c \| 2 \| 5 \| TTTGGTGTTAGCGCTGGAAGTATAGGGACAGAGTACTTGATAACATCATT \| 16.518 \| 25.067 \| 0 \| 0 \| 0 \| 3 \| 3 \| \| SPAC323.05c \| 2 \| 4 \| AAAACTTATTAACAGCGGAAATTGGTTCATTTTATGTCCGACATCTCAAA \| 13.92 \| 5.34 \| 0 \| 0 \| 2 \| 2 \| 4 \| \| SPAC17G8.14c \| 1 \| 3 \| AACAGTTTAGCGTGAAACGTGCAAGATTTTATTAGAACAGCAATATTCCA \| 13.835 \| 24.708 \| 0 \| 0 \| 4 \| 6 \| 10 \| \| SPBC31F10.13c \| 3 \| 5 \| TAGTTATTATATGGCACAAGGAGGAATATCCAGGATTTATGTTGGAGTTA \| 13.265 \| 5.368 \| 0 \| 0 \| 3 \| 0 \| 3 \| \| SPBC3H7.08c \| 1 \| 3 \| GTCAAGAGAATATCCAGAACTATTTGACAGCGGGGCTTGTTGGTACAACA \| 11.281 \| 33.033 \| 0 \| 0 \| 2 \| 1 \| 3 \| \| SPAC227.10 \| 1 \| 4 \| GCTGAACAACCTTCTCGGCAGCAGAATTCAAACAGCCATGAATGGTTTGC \| 10.335 \| 19.005 \| 0 \| 0 \| 1 \| 4 \| 5 \| \| SPAC824.09c \| 1 \| 3 \| ACTTGGGTGTTTTCATTTGTATCAGTAAAATCTGTTGATTTGGATTCATG \| 9.62 \| 11.269 \| 0 \| 1 \| 2 \| 1 \| 4 \| \| SPBC83.01 \| 1 \| 3 \| TCTTCCTCAAAAGATATTAGCAAAGCCGGAGATCCTCCAATTTTACCAAA \| 9.233 \| 13.673 \| 0 \| 0 \| 0 \| 5 \| 5 \| \| SPBC1711.03 \| 1 \| 3 \| ATGCAAAAGCTTCTGCTGGATCCAGACGACTACTACAGCGCGCTTATGCC \| 8.401 \| 8.057 \| 1 \| 1 \| 5 \| 4 \| 11 \| \| SPCC126.11c \| 1 \| 3 \| CGAGGCAACAAAAAGAAGCAAGAAGGGATCAGCTTTAACTTCAGATAAAA \| 7.938 \| 11.35 \| 0 \| 0 \| 3 \| 2 \| 5 \| \| SPAC630.11 \| 1 \| 4 \| AATTAAATATGTCCGACCTCAGGAATCATTCCATTCATTTTAGCTCCTCT \| 7.478 \| 7.607 \| 1 \| 0 \| 0 \| 5 \| 6 \| \| SPBC106.05c \| 1 \| 3 \| AAAAATGAATTCCATAAACGTAAAGAAGACTCTATGTTGCCTAATTTGAA \| 7.405 \| 7.463 \| 0 \| 0 \| 0 \| 4 \| 4 \| \| SPBC16H5.04 \| 1 \| 3 \| GGAGAAATTGAAGAATAATCCTCAAATTTAACTGTTTTAAAATATGTTGA \| 7.161 \| 5.673 \| 0 \| 0 \| 1 \| 2 \| 3 \| \| SPCC320.10 \| 2 \| 4 \| TTACGAACACCTTGAAAATGATTTGTCTAGACGAAACTTCTCAGGCAATC \| 7.042 \| 10.565 \| 0 \| 0 \| 0 \| 7 \| 7 \| \| SPCC622.14 \| 1 \| 3 \| TGAACGTCAAGTGAAGATGATGGAGTACAATACCGACATTGCTGAGGACC \| 6.672 \| 5.708 \| 0 \| 0 \| 3 \| 1 \| 4 \| \| SPBC1A4.08c \| 1 \| 3 \| ATGCAGTCTCCTGTGTTTGTTATGAGTTATTCGAACATGTTTAGGCCCGA \| 6.555 \| 15.986 \| 0 \| 0 \| 2 \| 1 \| 3 \| \| SPCC338.15 \| 2 \| 4 \| ATCTTATTGTCATCTCAAAGTAAAAATTTTCTCAATCGACATATCTCTTT \| 6.526 \| 7.743 \| 0 \| 0 \| 2 \| 1 \| 3 \| \| SPAC22G7.04 \| 2 \| 5 \| ATTAGTATGGACTGGACATAAAAATGACAAAAACGTTAGTGACATTTTCA \| 6.498 \| 5.757 \| 0 \| 0 \| 0 \| 4 \| 4 \| \| SPBC19G7.14c \| 1 \| 3 \| AGGGATCGATTAATGAAAATGAAAAAAAAGACAATCGTCCGTTTCTTTAC \| 6.459 \| 5.365 \| 0 \| 0 \| 0 \| 3 \| 3 \| \| SPAC1639.02c \| 1 \| 7 \| CACGCAGATATCCATTTACCAACAGGAACGTTCTCTTGGAATATATGCAG \| 6.071 \| 5.659 \| 0 \| 0 \| 2 \| 3 \| 5 \| \| SPAC1093.03 \| 1 \| 5 \| ATTCGAGCTGTACCAGCTAAAAAAAAAAAGTTCCTGTGACTAAAACTCTA \| 5.535 \| 5.945 \| 0 \| 0 \| 0 \| 3 \| 3 \| \| SPAC17G6.16c \| 3 \| 6 \| TTTTATCTGACTACGTGAAAGTGAGTGACGAGCCATAAATGTAACCATTT \| 5.534 \| 5.44 \| 0 \| 0 \| 0 \| 3 \| 3 \| \| SPCC18B5.11c \| 2 \| 4 \| TCTGAGAATGTCGTTTTTTTACACGGAACGGCTTGCCAAGAATAGTAGAA \| 5.221 \| 7.348 \| 0 \| 0 \| 2 \| 2 \| 4 \| \| SPAC3A11.06 \| 2 \| 6 \| GAACAGATTTTCAAGCAAAGAAATGCGAAATGCTTCAACTCAAAACAGTT \| 5.216 \| 5.746 \| 0 \| 0 \| 6 \| 10 \| 16 \| \| SPBC1709.02c \| 1 \| 3 \| AGGCTGTGAAGCTGCTCAGTCTAAGTAGAGCGTGAACGCCAAAAGGCGGC \| 5.189 \| 9.461 \| 0 \| 0 \| 2 \| 5 \| 7 \| \| SPBC337.09 \| 1 \| 3 \| CGTGTATATAGCAACACTAACGAAGTGTGTATTTTCTATGCCAATGCACA \| 5.181 \| 7.229 \| 0 \| 0 \| 17 \| 10 \| 27 \| \| SPAC630.03 \| 1 \| 3 \| TTAATGTTCCCATTATAATGGACAACTATGCTGGGAACGATGCTCCTTCT \| 5.108 \| 5.38 \| 0 \| 0 \| 2 \| 4 \| 6 \| \| SPAC23H4.04 \| 1 \| 3 \| CAATGGCCCAAATCGCAGGATAAAGGGATATAATGTGGAAGGTGTTTTTA \| 5.084 \| 8.387 \| 0 \| 0 \| 2 \| 2 \| 4 \| \| SPBC17D1.07c \| 1 \| 4 \| TTTGTAGCGTTCATGAAGCTAAAAAGAATCTTTTAAGCCTTCAGCTATTA \| 5.065 \| 5.49 \| 0 \| 0 \| 2 \| 1 \| 3 \| \| SPAC4G8.11c \| 1 \| 3 \| TCCTCCAAGATTATTTAAGGAGAAGAATGAATATATTACAGCATACGACA \| 5.053 \| 8.035 \| 0 \| 0 \| 0 \| 0 \| 5 \| \| SPBC660.16 \| 1 \| 5 \| GAAGAATCATGTCACAAAAAGAAGTGTAAATCTATTGTTGGTGCTCACTC \| 4.982 \| 12.521 \| 0 \| 0 \| 0 \| 4 \| 4 \| \| SPBC21.02 \| 1 \| 3 \| GCTTTAGAACGGTATAATTTAAAAGCACGACGTTCTTTGGAATTCCAGAC \| 4.975 \| 5.498 \| 0 \| 0 \| 0 \| 4 \| 4 \| \| SPBC839.17c \| 1 \| 4 \| GGGTGTCGAAAAGCAAGTTATTTCTGTACTTTAACCAATGGTAAAAAGTT \| 4.94 \| 5.514 \| 0 \| 0 \| 1 \| 2 \| 3 \| \| SPAC9G1.13c \| 1 \| 4 \| AAGTTAGTCACAAAGCTAAAAATTGATGAGGATTGGAATAAAGATGAAAC \| 4.93 \| 5.388 \| 0 \| 0 \| 8 \| 0 \| 8 \| \| SPBC19C7.10 \| 1 \| 3 \| AAAGCAGGTCTTTACCTGCTGAAAGATACACCATTAATCCCAAAGTGTCG \| 4.924 \| 6.694 \| 0 \| 0 \| 6 \| 4 \| 10 \| \| SPCC126.06 \| 1 \| 3 \| ATGTCTGCTTCCGTCGAATTAAAACAGAATTCATTTGACGTGAAAACAAT \| 4.904 \| 8.978 \| 0 \| 0 \| 5 \| 3 \| 8 \| \| SPAC3A11.06 \| 4 \| 6 \| CCTTTTAGAAGAGTACCATTAATACCGAAATGCTTCAACTCAAAACAGTT \| 4.87 \| 5.355 \| 0 \| 0 \| 4 \| 0 \| 4 \| \| SPAC12B10.05 \| 4 \| 7 \| ATGAATACCACCAGGATCCAAATTTTTAAAAATGGAGCAAGTGGAAGTTA \| 4.845 \| 12.819 \| 0 \| 0 \| 1 \| 2 \| 4 \| \| SPBC725.08 \| 2 \| 4 \| AATTACTATGATGATAGAACAAGAGTTTAATTGATCCATATACTCAAACA \| 4.82 \| 5.35 \| 0 \| 0 \| 3 \| 0 \| 3 \| \| SPAC15E1.09 \| 1 \| 3 \| AAGGCTTATGAGTTAGATAAAATAGCTTATTTACATGAAAAGACCAAACA \| 4.81 \| 5.394 \| 0 \| 0 \| 4 \| 13 \| 17 \| \| SPAC4F10.20 \| 1 \| 4 \| AACCATGTCTAGTGTTGAATCATTTCTTACTTATTGAAGAAGACCGGTCA \| 4.78 \| 7.402 \| 0 \| 0 \| 0 \| 7 \| 7 \| \| SPAC30C2.05 \| 1 \| 3 \| CTTTTGTTTACATAGCATGTCTTTTGATTTAGAGATGGACTATATTAACC \| 4.755 \| 6.372 \| 0 \| 0 \| 1 \| 3 \| 4 \| \| SPAC630.11 \| 1 \| 5 \| AATTAAATATGTCCGACCTCAGGAAAAATCTTCTTGACTTTGGTCGATTT \| 4.665 \| 5.27 \| 0 \| 0 \| 3 \| 9 \| 12 \| |  |  |  |  |  |  |  |  |  |  |
| --- | --- | --- | --- | --- | --- | --- | --- | --- | --- | --- | --- | --- | --- | --- | --- | --- | --- | --- | --- | --- | --- | --- | --- | --- | --- | --- | --- | --- | --- | --- | --- | --- | --- | --- | --- | --- | --- | --- | --- | --- | --- | --- | --- | --- | --- | --- | --- | --- | --- | --- | --- | --- | --- | --- | --- | --- | --- | --- | --- | --- | --- | --- | --- | --- | --- | --- | --- | --- | --- | --- | --- | --- | --- | --- | --- | --- | --- | --- | --- | --- | --- | --- | --- | --- | --- | --- | --- | --- | --- | --- | --- | --- | --- | --- | --- | --- | --- | --- | --- | --- | --- | --- | --- | --- | --- | --- | --- | --- | --- | --- | --- | --- | --- | --- | --- | --- | --- | --- | --- | --- | --- | --- | --- | --- | --- | --- | --- | --- | --- | --- | --- | --- | --- | --- | --- | --- | --- | --- | --- | --- | --- | --- | --- | --- | --- | --- | --- | --- | --- | --- | --- | --- | --- | --- | --- | --- | --- | --- | --- | --- | --- | --- | --- | --- | --- | --- | --- | --- | --- | --- | --- | --- | --- | --- | --- | --- | --- | --- | --- | --- | --- | --- | --- | --- | --- | --- | --- | --- | --- | --- | --- | --- | --- | --- | --- | --- | --- | --- | --- | --- | --- | --- | --- | --- | --- | --- | --- | --- | --- | --- | --- | --- | --- | --- | --- | --- | --- | --- | --- | --- | --- | --- | --- | --- | --- | --- | --- | --- | --- | --- | --- | --- | --- | --- | --- | --- | --- | --- | --- | --- | --- | --- | --- | --- | --- | --- | --- | --- | --- | --- | --- | --- | --- | --- | --- | --- | --- | --- | --- | --- | --- | --- | --- | --- | --- | --- | --- | --- | --- | --- | --- | --- | --- | --- | --- | --- | --- | --- | --- | --- | --- | --- | --- | --- | --- | --- | --- | --- | --- | --- | --- | --- | --- | --- | --- | --- | --- | --- | --- | --- | --- | --- | --- | --- | --- | --- | --- | --- | --- | --- | --- | --- | --- | --- | --- | --- | --- | --- | --- | --- | --- | --- | --- | --- | --- | --- | --- | --- | --- | --- | --- | --- | --- | --- | --- | --- | --- | --- | --- | --- | --- | --- | --- | --- | --- | --- | --- | --- | --- | --- | --- | --- | --- | --- | --- | --- | --- | --- | --- | --- | --- | --- | --- | --- | --- | --- | --- | --- | --- | --- | --- | --- | --- | --- | --- | --- | --- | --- | --- | --- | --- | --- | --- | --- | --- | --- | --- | --- | --- | --- | --- | --- | --- | --- | --- | --- | --- | --- | --- | --- | --- | --- | --- | --- | --- | --- | --- | --- | --- | --- | --- | --- | --- | --- | --- | --- | --- | --- | --- | --- | --- | --- | --- | --- | --- | --- | --- | --- | --- | --- | --- | --- | --- | --- | --- | --- | --- | --- | --- | --- | --- | --- | --- | --- | --- | --- | --- | --- | --- | --- | --- | --- | --- | --- | --- | --- | --- | --- | --- | --- | --- | --- | --- | --- | --- | --- | --- | --- | --- | --- | --- | --- | --- | --- | --- | --- | --- | --- | --- | --- | --- | --- | --- | --- | --- | --- | --- | --- | --- | --- | --- | --- | --- | --- | --- | --- | --- | --- | --- | --- | --- | --- | --- | --- | --- | --- | --- | --- | --- | --- | --- | --- | --- | --- | --- | --- | --- | --- | --- | --- | --- | --- | --- | --- | --- | --- | --- | --- | --- | --- | --- | --- | --- | --- | --- | --- | --- | --- | --- | --- | --- | --- | --- | --- | --- | --- | --- | --- | --- | --- | --- | --- | --- | --- | --- | --- | --- | --- | --- | --- | --- | --- | --- | --- | --- | --- | --- | --- | --- | --- | --- | --- | --- | --- | --- | --- | --- | --- | --- | --- | --- | --- | --- | --- | --- | --- | --- | --- | --- | --- | --- | --- | --- | --- | --- | --- | --- | --- | --- | --- | --- | --- | --- | --- | --- | --- | --- | --- | --- | --- | --- | --- | --- | --- | --- | --- | --- | --- | --- | --- | --- | --- | --- | --- | --- | --- | --- | --- | --- | --- | --- | --- | --- | --- | --- | --- | --- | --- | --- | --- | --- | --- | --- | --- | --- | --- | --- | --- | --- | --- | --- | --- | --- | --- | --- | --- | --- | --- | --- | --- | --- | --- | --- | --- | --- | --- | --- | --- | --- | --- | --- | --- | --- | --- | --- | --- | --- | --- | --- | --- | --- | --- | --- | --- | --- | --- | --- | --- | --- | --- | --- | --- | --- | --- | --- | --- | --- | --- | --- | --- | --- | --- | --- | --- | --- | --- | --- | --- | --- | --- | --- | --- | --- | --- | --- | --- | --- | --- | --- | --- | --- | --- | --- | --- | --- | --- | --- | --- | --- | --- | --- | --- | --- | --- | --- | --- | --- | --- | --- | --- | --- | --- | --- | --- | --- | --- | --- | --- | --- | --- | --- | --- | --- | --- | --- | --- | --- | --- | --- | --- | --- | --- | --- | --- | --- | --- | --- | --- | --- | --- | --- | --- | --- | --- | --- | --- | --- | --- | --- | --- | --- | --- | --- | --- | --- | --- | --- | --- | --- | --- | --- | --- | --- | --- | --- | --- | --- | --- | --- | --- | --- | --- | --- | --- | --- | --- | --- | --- | --- | --- | --- | --- | --- | --- | --- | --- | --- | --- | --- | --- | --- | --- | --- | --- | --- | --- | --- | --- | --- | --- | --- | --- | --- | --- | --- | --- | --- | --- | --- | --- | --- | --- | --- | --- | --- | --- | --- | --- | --- | --- | --- | --- | --- | --- | --- | --- | --- | --- | --- | --- | --- | --- | --- | --- | --- | --- | --- | --- | --- | --- | --- | --- | --- | --- | --- | --- | --- | --- | --- | --- | --- | --- | --- | --- | --- | --- | --- | --- | --- | --- | --- | --- | --- | --- | --- | --- | --- | --- | --- | --- | --- | --- | --- | --- | --- | --- | --- | --- | --- | --- | --- | --- | --- | --- | --- | --- | --- | --- | --- | --- | --- | --- | --- | --- | --- | --- | --- | --- | --- | --- | --- | --- | --- | --- | --- | --- | --- | --- | --- | --- | --- | --- | --- | --- | --- | --- | --- | --- | --- | --- | --- | --- | --- | --- | --- | --- | --- | --- | --- | --- | --- | --- | --- | --- | --- | --- | --- | --- | --- | --- | --- | --- | --- | --- | --- | --- | --- | --- | --- | --- | --- | --- | --- | --- | --- | --- | --- | --- | --- | --- | --- | --- | --- | --- | --- | --- | --- | --- | --- | --- | --- | --- | --- | --- | --- | --- | --- | --- | --- | --- | --- | --- | --- | --- | --- | --- | --- | --- | --- | --- | --- | --- | --- | --- | --- | --- | --- | --- | --- | --- | --- | --- | --- | --- | --- | --- | --- | --- | --- | --- | --- | --- | --- | --- | --- | --- | --- | --- | --- | --- | --- | --- | --- | --- | --- | --- | --- | --- | --- | --- | --- | --- | --- | --- | --- | --- | --- | --- | --- | --- | --- | --- | --- | --- | --- | --- | --- | --- | --- | --- | --- | --- | --- | --- | --- | --- | --- | --- | --- | --- | --- | --- | --- | --- | --- | --- | --- | --- | --- | --- | --- | --- | --- | --- | --- | --- | --- | --- | --- | --- | --- | --- | --- | --- | --- | --- | --- | --- | --- | --- | --- | --- | --- | --- | --- | --- | --- | --- | --- | --- | --- | --- | --- | --- | --- | --- | --- | --- | --- | --- | --- | --- | --- | --- | --- | --- | --- | --- | --- | --- | --- | --- | --- | --- | --- | --- | --- | --- | --- | --- | --- | --- | --- | --- | --- | --- | --- | --- | --- | --- | --- | --- | --- | --- | --- | --- |
|  |  |  |  |  |  |  |  |  |  |  |
