# Supplementary material for: Functions for fission yeast splicing factors SpSlu7 and SpPrp18 in alternative splice-site choice and stress-specific regulated splicing
Source: PLoS One. 2017 Dec 13;12(12):e0188159. doi: 10.1371/journal.pone.0188159 (PMC5728500; doi:10.1371/journal.pone.0188159)
Supplement: S3 Table — In order to detect the ten isoforms formed due to the utilisation of altered 5’ss (donors) or 3’ss (acceptors) each, as reported in [16], twenty probe sequences were custom designed and incorporated in the SpPrp18 microarray platform [32]. (DOCX) [file pone.0188159.s010.docx]

**S3 Table. List of twenty custom-designed probes to detect alternate mRNA isoforms arising from altered use of splice sites.** In order to detect the ten isoforms formed due to the utilisation of altered 5’ss (donors) or 3’ss (acceptors) each, as reported in [16], twenty probe sequences were custom designed and incorporated in the SpPrp18 microarray platform [32].

| **Probe_id** | **Sequence (5'- 3')** |
| --- | --- |
| **ALTERNATE ACCEPTOR PROBES** | |
| **SPAC17C9.11c\|EX1-EX2_1_accept** | **TCAATTTGAGGAAACTGAAGAAGAGGTTCGAATTGTTAACCATCCTTATT** |
| **asmbl_3133_accept** | **ACTTCAGGTGCAACAGGAGCAACAGTGGTTGAGGAACTGAAGGAACTTCA** |
| **SPAC23H4.02\|EX1-EX2_1_accept** | **GGGCTTGTTAATAAGATAGAGTAGAACAAGGAAACTTGGCCAAAGTGAAA** |
| **asmbl_7672_accept** | **CGAAAAGTTTACGTTAATTTTATCGAAATGGGGAGAAGCAGCAAAGATAA** |
| **SPCC1281.08\|EX1-EX2_1_accept** | **GAGAGCGGTTTGCCTTCAAATTCAGTTCTTCATATTTAATCAATCCTGAT** |
| **SPCC18.09c\|EX1-EX2_1_accept** | **AAAACTTATTGGATATTCAGTATACGAGCGTACACAAGACGAATGATGCT** |
| **asmbl_14785_accept** | **TTTCATCAAGGTAATTAACGAATGTGTTTGTAAGACGGTTTTGCAACACA** |
| **SPBC3E7.04c\|EX1-EX2_1_accept** | **TACAGATTCTAAATGAAAAAGGCCTCATCAAAAGTAGTTGCAAAATCTTT** |
| **SPBC577.11\|EX1-EX2_1_accept** | **TACACGTTCCCTGAAAACTTTCAAGAAGGATTGCTATACGAACATATCAA** |
| **asmbl_18393_accept** | **GCCCTGAATTTAGCTACAAACTATGATAAAAATTGCTTAAACTGTTTACT** |
| **ALTERNATE DONOR PROBES** | |
| **SPAC1399.05c\|EX1_1-EX2_donor** | **TTCCTCTTACTGTAGTCTTGTTGTTCTCAAGGTATTGGAGAAGGCCATCA** |
| **SPAC8C9.05\|EX1_1-EX2_donor** | **TAAGTTTGGACGGATTTTATAATCGTGATGACAAAATTGTTTCTGCCATT** |
| **asmbl_2356_donor** | **CCTGATGAAGTGAAGGTGCTAGTGGAGCAACGGTGGAGTTGCTCAATGGA** |
| **asmbl_5336_donor** | **ACGTTGTAATGTCACTTTCAAAGAATCAGCAATACTGAACTTCACATATC** |
| **asmbl_8229_donor** | **ACATGTAATCATCTTCCACTTTGTACGAGAGTTTCACACAATTTCGCCCA** |
| **SPCC965.03.1\|EX2_1-EX3_donor** | **TTTACAAATCTACTGTGTATTATAGCAAAACAAAAGATGGGCCGTGTGAT** |
| **asmbl_14785_donor** | **TTTCATCAAGGTAATTAACGAATGTGTTTGTAAGACGGTTTTGCAACACA** |
| **SPBC25B2.06c\|EX1_1-EX2_donor** | **ATTTCAAGTTTCGTATTTAATGCAGTGTTTAACGATTTCCAAACAAAAA** |
| **asmbl_16630_donor** | **TAATATTAGGCTGGATGTTTCTGATTTATCAAATCGATTTCCACTCACGA** |
| **SPBC19C2.08.1\|EX1_1-EX2_donor** | **TGGCAAGCAAAGAAACCAAGTATAGTTGTTCATTGGTGGATCGTGCAGTA** |
